# Effects of Electron-Withdrawing Strengths of the Substituents on the Properties of 4-(Carbazolyl-*R*-benzoyl)-5-CF_3_-1*H*-1,2,3-triazole Derivatives as Blue Emitters for Doping-Free Electroluminescence Devices

**DOI:** 10.1021/acsomega.4c01077

**Published:** 2024-03-12

**Authors:** Mariia Stanitska, Nazariy Pokhodylo, Roman Lytvyn, Ervinas Urbonas, Dmytro Volyniuk, Stepan Kutsiy, Khrystyna Ivaniuk, Vasyl Kinzhybalo, Pavlo Stakhira, Rasa Keruckiene, Mykola Obushak, Juozas Vidas Gražulevičius

**Affiliations:** † Kaunas University of Technology, Baršausko st. 59, 51423 Kaunas, Lithuania; ‡ Ivan Franko National University of Lviv, Kyryla i Mefodiya 6, Lviv 79005, Ukraine; § National University “Lviv Polytechnic”, Stepan Bandera 12, Lviv 79000, Ukraine; ∥ Institute of Low Temperature and Structure Research, Okólna 2, Wrocław 50-422, Poland

## Abstract

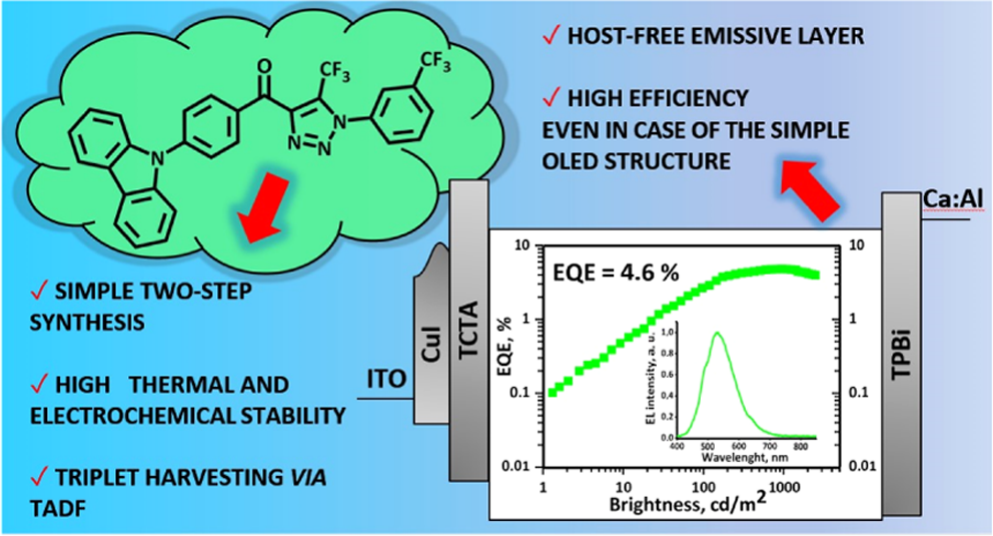

The synthesis of four 4-(carbazolyl-*R*-benzoyl)-5-CF_3_-1*H*-1,2,3-triazoles with extra groups ((3-methyl)-phenyl-, 4-fluorophenyl-, quinolinyl-, or (3-trifluoromethyl)-phenyl-) in the acceptor fragment has been reported. The effects of substituents with different electron-withdrawing strengths on the thermal, electrochemical, photophysical, and electroluminescence properties of the synthesized compounds are discussed. The results of X-ray analyses and density functional theory (DFT) calculations support unusual molecular packing and electronic properties. The compounds are capable of glass formation with glass transition temperatures ranging from 54–84 °C. Ionization potentials of the compounds are in the range of 5.98–6.22 eV and electron affinities range from 3.09 to 3.35 eV. Under ultraviolet excitation, the neat films of the compounds exhibit blue emission with photoluminescence quantum yields ranging from 18 to 27%. The films of selected compounds are used for the preparation of host-free light-emitting layers of organic light-emitting diodes with very simple device structures and an external quantum efficiency of 4.6%.

## Introduction

Studies of organic emitters exhibiting thermally activated delayed fluorescence (TADF) have taken a giant leap forward since the first observation of this phenomenon in 1961.^1^ Despite the rapid and profound evolution of the practical branch of this concept since 2012, TADF still attracts attention of researchers due to the possibility to harvest “dark” triplet excitons through the reverse intersystem crossing and thus to achieve 100% of internal quantum efficiency of organic light-emitting diodes (OLEDs).^2^ The state-of-art TADF-based OLEDs are fabricated using complicated multilayered structures containing many functional layers. They are typically based on guest–host/cohost systems with a specific combination of the electronic properties of guests and hosts.^3,4^ However, efficient TADF-based OLEDs with host-free emitting layers are relatively rare due to the limited number of emitters with a perfect combination of required electronic properties. In particular, in the case of blue OLEDs, device efficiencies decrease considerably when one or more functional organic layers are skipped from the OLED structures.^5^ A notable example is yellow single-layer TADF-based OLEDs with an external quantum efficiency (EQE) of 19%.^6^ Such OLEDs were fabricated with a single layer of 9,10-bis(4-(9*H*-carbazol-9-yl)-2,6-dimethylphenyl)-9,10-diboraanthracene, which was characterized by high photoluminescence quantum yield, and energy levels ensuring the barrier-free transport of holes and electrons. In this work, we aimed to obtain a similar combination of electronic properties of the new compounds but with emission in the blue spectral region. The molecular design of TADF emitters is unimaginable without nitroaromatic compounds.^7^ The determining factor that specifies the electronic properties of such heterocycles is the location of free nonbonding electrons on nitrogen atoms. In the case of an essential electron-donor 9*H*-carbazole, the electron pair on the nitrogen atom participates in electron delocalization, inducing the appearance of negative charges on carbon atoms, thus bearing the electron-donating ability of the compound.^8^ In contrast, the lone electron pairs of nitrogen atoms of pyrimidine and triazine are in the equatorial position to the ring that constrains them from being involved in π-conjugation and causes the appearance of partly positive charges on carbon atoms.^9,10^ This makes them favorable acceptor constituents in the design of donor–acceptor (D–A)-type electroactive compounds. Moreover, in nitrogen-containing heterocycles, triplet states with *n*π* character are present, which facilitates intersystem crossing, thus increasing the intensity of delayed fluorescence.^11^ Pyridine, a six-membered heterocycle, gained widespread application in the design of D–A-type TADF emitters, especially when the ring is decorated with strong electron-withdrawing substituents such as carbonitrile.^12^ Pyridine-containing materials are among the first-rate sky-blue emitters for OLEDs, which demonstrate EQE close to 30%.^13^ Green and yellow emitting pyridine-based OLEDs exhibited remarkable EQEs of 30.3 and 29.2% respectively.^14^ A strong electron acceptor triazine moiety, which has three nitrogen and three carbon atoms alternating in the aromatic ring, was combined with the spiro-biacridine electron-donor unit in order to achieve considerable steric hindrance and high PLQYs of the compound in the solid state.^15^ EQEs of nearly 21% were obtained for pure blue OLEDs, close to 30% for sky-blue devices, and over 35% for greenish-blue devices.^16^ Unsymmetrical triazine-acridine conjugate that also had a quinoline moiety in the structure was reported.^17^ In this molecule, double charge-transfer excited states were observed. The solution of the compound displayed dual emission, consisting of dominant orange-red emission and sky-blue emission. An extremely high EQE of 31.7% was achieved for OLEDs, with an electroluminescence peak at 593 nm.^17^

Triazole is a five-membered heterocyclic aromatic system having three nitrogen atoms. The geometry of the electron pair of the singly bonded nitrogen atom is pyrrole-like, while two others demonstrate pyridine-like behavior.^18^ This leads to electron-accepting character which is lower than that of pyridine and triazine but can be significantly reinforced by functionalizing the triazole cycle with various electron-withdrawing substituents.^19^ In addition, the decoration of the triazole ring with phenyl substituents through nitrogen atoms allows the achievement of efficient luminescence and high photostability.^20^ The values of triplet energy levels above 3 eV allow triazole derivatives to be used as appropriate building blocks for the design of TADF materials.^21,22^ Meanwhile, the potential of triazole-based materials as promising TADF emitters is rather neglected. Fluorescent OLEDs based on 4*H*-1,2,4-triazole derivatives with a maximum EQE of 6.3% and exceptional color purity were reported.^23^ According to DFT calculations, in this case, the highest occupied molecular orbital (HOMO) is spread over both triazole rings and electron-donor fragments, which is the restriction for TADF.^23^ Triazoles have gained more widespread application as hosts and electron-transporting materials.^24−26^ Bistriazole derivatives with comparable electron and hole mobilities and a large bandgap of 4.0 eV were reported as hosts for blue phosphorescent OLEDs, which demonstrated an EQE exceeding 30%.^27^ Bipolar hosts demonstrating TADF consisting of triazole as the acceptor and dimethylacridine as the donor were developed.^28^ The TADF host achieved an EQE of 13.5% with a low roll-off value of 4.4% at a luminance of 1000 cd/m^2^. In our previous work, we reported the convenient synthesis of 1*H*-1,2,3- triazole-based electroactive materials exhibiting TADF.^29^ They were used as hosts in efficient solution-processed hybrid white light-emitting diodes with a multilayer structure.^29^ However, to our knowledge, 1*H*-1,2,3-triazole derivatives have not yet been used for the preparation of host-free emitting layers for OLEDs.

To identify blue emitters for simplified doping-free electroluminescence devices, we synthesized four new 1*H*-1,2,3-triazole-cored D–A compounds. The electronic properties of the compounds were modified by the attachment of extra groups to the acceptor fragments ((3-methyl)-phenyl-, 4-fluorophenyl-, quinolinyl-, or (3-trifluoromethyl)-phenyl) with different electron-withdrawing strengths. The effects of the attachment of these groups on the thermal, electrochemical, photophysical, and electroluminescence properties of the synthesized compounds were studied using theoretical and experimental approaches, including density functional theory (DFT) calculations, X-ray analysis, and optical and luminescence spectroscopy. The best result was achieved for 4-((9*H*-carbazol-9-yl)phenyl)(1-(m-tolyl)-5-(trifluoromethyl)-1*H*-1,2,3-triazol-4-yl)methanone containing a (3-trifluoromethyl)-phenyl- group. This compound was used as the light-emitting material in the simplified OLEDs, which showed a maximum EQE of up to 4.6%. The fabricated OLEDs exhibited a greenish-blue emission. Their EL spectra were characterized by Commission International de l′Eclairage (CIE) coordinates of (0.34, 0.46).

## Results and Discussion

### Synthesis and X-ray Analysis

**Figure 1:**
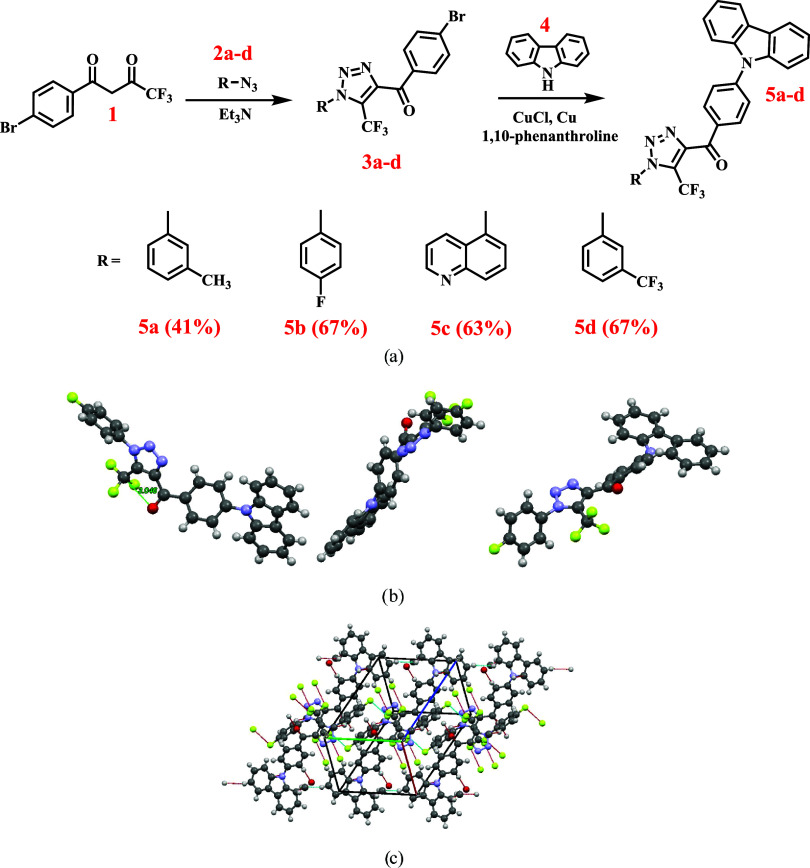
General scheme for the synthesis of compounds **5a**–**c**; yields are shown in parentheses (a). X-ray crystal structure of compound **5b** and schematic views from different perspectives (b). Arrangement of molecules of **5b** in crystal (c).

Following the above-described idea of molecular design, donor–acceptor triazole-based compounds **5a**–**d** were obtained via a three-step synthetic pathway (Figure 1a). The building block for the formation of the triazole ring, 1,3-diketone **1** was easily synthesized from commercially available 4′-bromoacetophenone via Claisen condensation with ethyl trifluoroacetate. The treatment of compound **1** with the appropriate azide **2a**–**d** in trimethylamine resulted in the formation of the corresponding triazoles **3a**–**d** in 79–88% yields.

Carbazolyl-substituted triazoles 5a–d were obtained via copper-catalyzed Ullmann–Goldberg cross-coupling of carbazole **4** and the corresponding brominated derivatives **3a**–**d**. Refluxing a mixture of **3a**–**d**, carbazole, copper, copper chloride, potassium carbonate, and phenanthroline in xylene afforded the target compounds **5a**, **5b**, **5c**, and **5d**. Although the reaction conditions were rather harsh, the products of the nucleophilic substitution of fluorine atoms were not detected. ^1^H and ^13^C NMR spectroscopy, elemental analysis, and mass spectrometry were used to identify the chemical structures of the compounds (Supporting Information).

X-ray single-crystal analysis was performed for compound **5b**^1^. The crystals were grown by slow evaporation of ethanol from the solution. Figure 1b, **c** represents the conformational pattern and the packing of the molecules of **5b** in a single crystal. Single-crystal data analysis revealed that compound **5b** crystallized in a triclinic crystal system in the space group *P*1̅ (Table S1). In the crystalline lattice, compound **5b** takes part in abundant intermolecular interactions of several types, such as π···π stacking of adjacent carbazolyl units with a distance of 3.391 Å; C–H···π interactions between adjacent carbazolyl units with a distance of 2.715 Å; C–H···O interactions between the adjacent benzoyl units with a distance of 2.449 Å; F···F interactions with a distance of 2.924 Å between neighboring trifluoromethyl fragments; and C–F···F interactions between the 4-fluorophenyl fragment and the neighboring fluorine atom from the trifluoromethyl group with a distance of 2.875 Å. According to the X-ray data, trifluoromethyl and carbonyl groups, which both express strong electron-accepting characteristics are located in close proximity to each other, although the rotation around the carbonyl-triazole bond is not restricted. The distance between them is estimated to be 3.046 Å. Such uncommon packing can be explained by the presence of a partly positive charge on the carbon atom of the trifluoromethyl group and the presence of a lone electron pair on the oxygen atom of the carbonyl group, resulting in attractive CF_3_–O interactions. Wildberg et al. first theoretically investigated such kind of interactions in 2019.^30^ According to the reported results, the theoretically predicted distance between the carbon atom of the trifluoromethyl group and the oxygen atom of the keto group in the complex of CF_4_ and acetone is 3.23 Å. In the case of compound **5b**, such a distance is even shorter, resulting in a higher interaction energy.

### Theoretical Calculations and Electrochemical Properties

**Figure 2:**
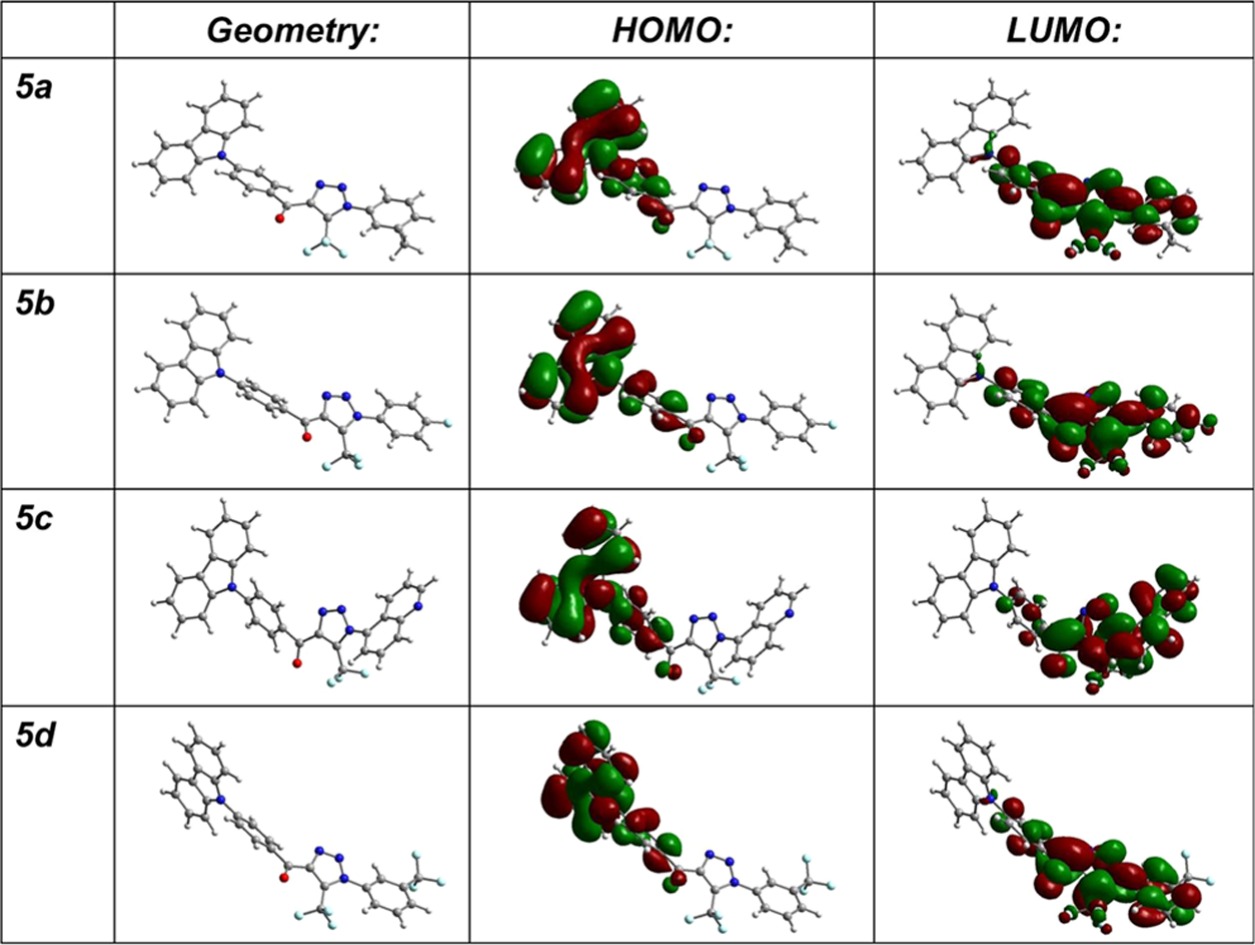
Optimized geometries at the ground state and distributions of HOMO/LUMO orbitals of compounds **5a**–**5d** obtained by theoretical calculations.

Visualization of the optimized ground-state (S_0_) geometries demonstrates that the structures of all of the compounds are not planar, and central phenyl rings are rotated by 44° with respect to the carbazole scaffolds (Figure 2). Such values of the dihedral angles are typical for the molecules of carbazole derivatives with a donor–acceptor architecture.^31,32^

Due to the similarity of the electronic properties of the donor and acceptor constituent fragments and the similarity of the optimized geometries, the delocalization of the frontier molecular orbitals (FMOs) is almost the same for all four compounds. No shift in the FMO distribution was observed with the change of acceptor strength. The HOMOs were found to be distributed mainly over the electron-donating phenylcarbazole fragments, whereas the lowest unoccupied molecular orbitals (LUMOs) were delocalized over the electron-accepting N-substituted triazole scaffolds. The HOMOs and LUMOs were not completely separated. Partial overlapping of the FMOs was also observed on the carbonyl groups and phenyl linkers for all of the studied compounds (Figure 2). Such a distribution of electronic density indicates the possibility of intramolecular charge transfer (ICT) from the carbazole donor to the triazole acceptor moiety. The energy levels of the HOMO and LUMO were found to be in the range of 5.53–5.45 and 2.38–2.65 eV, respectively. Compound **5d** was characterized by the deepest HOMO and LUMO levels of 5.35 and 2.65 eV, respectively (Table 1). Theoretical calculations demonstrated that triazole derivatives **5a**–**5d** can be regarded as promising CT emitters for OLEDs.

**Table 1:** Electrochemical Characteristics of Compounds **5a**–**c^d^**

	*E*_onset_^ox^, V	IP^CV^^a^, eV	*E*_onset_^red^, V	EA^CV^^b^, eV	*E*_G_^CV^^c^, eV	HOMO, eV	LUMO, eV	Δ_|HOMO–LUMO|_, eV
**5a**	0.88	5.98	–2.01	3.09	2.89	–5.45	–2.38	3.07
**5b**	0.94	6.04	–1.98	3.12	2.92	–5.49	–2.47	3.02
**5c**	0.96	6.06	–1.94	3.16	2.90	–5.46	–2.61	2.85
**5d**	1.12	6.22	–1.75	3.35	2.87	–5.53	–2.65	2.88

a Ionization potential was calculated using the equation IP^CV^ = 5.1 eV + *E*_ox_; *E*_onset_^red^ is the onset of the first reduction wave (with respect to ferrocene).

b Electron affinity was calculated using the equation EA^CV^ = 5.1 eV – *E*_red_.

c Energy gap was determined from the equation *E*_G_^CV^ = IP – EA.

d *E*_onset_^ox^ is the onset of the first oxidation wave (with respect to ferrocene).

**Figure 3:**
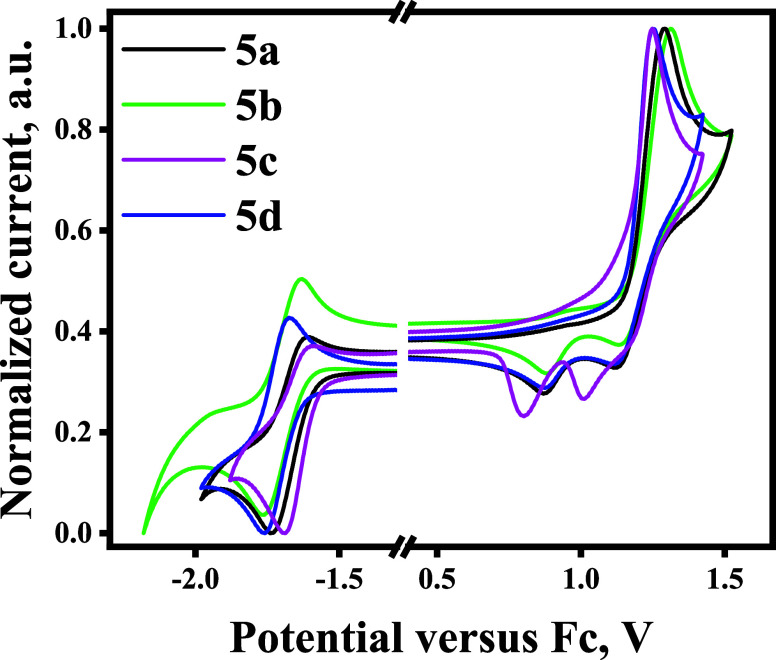
Cyclic voltammograms of compounds **5a**–**d**.

To estimate the electrochemical properties of compounds **5a**–**d**, cyclic voltammetry measurements were performed for dichloromethane solutions. Tetra-*n*-butylammonium hexafluorophosphate (TBAPF_6_) was used as a supporting electrolyte. All of the compounds were found to be electrochemically stable, as they all demonstrated reversible oxidation and reduction processes (Figure 3).

The ionization potential (IP^CV^) and electron affinity (EA^CV^) values as well as the corresponding onset potentials of the oxidation and reduction curves with respect to ferrocene are summarized in Table 1. Compound **5a**, which contains electron-donating methyl groups attached to the acceptor fragment, demonstrated the lowest IP of 5.98 eV. The IP^CV^ values of compounds **5b** and **5c** were found to be almost identical. One extra trifluoromethyl group in the structure of compound **5d** raised its ionization potential to 6.22 eV. This was the highest value obtained for the investigated series of compounds. The values of EA^CV^ showed a tendency to increase in the series of compounds from **5a** to **5d** with the change of the substituent at the first position of the triazole ring to a more electron-deficient one. Compound **5a**, which had the lowest electron-withdrawing ability of the acceptor moiety, demonstrated the lowest value of EA. The deepest EA was observed for compound **5d** due to the strong electron-withdrawing character of the two CF_3_ groups. The HOMO and IP^CV^ values were found to have a good correlation. The absolute IP^CV^ values were found to be slightly higher than the theoretically calculated HOMO energies. Compound **5d** exhibited the highest IP value of 6.22 eV and the deepest HOMO of −5.53 eV. Compounds **5b** and **5c** exhibited almost identical absolute values of IP^CV^ and HOMO energies of 6.06/–5.49 and 6.04/–5.46 eV. For compounds **5a** and **5b**, the LUMO energy values were found to be −2.38 and −2.42 eV, respectively. For quinolinyl- and (3-trifluoromethyl)-phenyl-substituted triazoles **5c** and **5d**, the LUMO energy values were deeper, i.e., of −2.61 and −2.65 eV. The EA^CV^ values electrochemically estimated for these compounds also correlated with the results obtained by theoretical calculations.

According to the results of DFT calculations, compound **5a** had the broadest energy bandgap of 2.92 eV. The broadest bandgap calculated from the CV data of *E*_G_^CV^ was observed for compound **5b** (2.92 eV). Nevertheless, both theoretically and experimentally estimated differences in the bandgaps for compounds **5c**–**5d** did not vary noticeably. This allows us to predict that all of the compounds could undergo effective charge transfer upon electrical excitation (Table 1).

### Photophysical Properties

**Figure 4:**
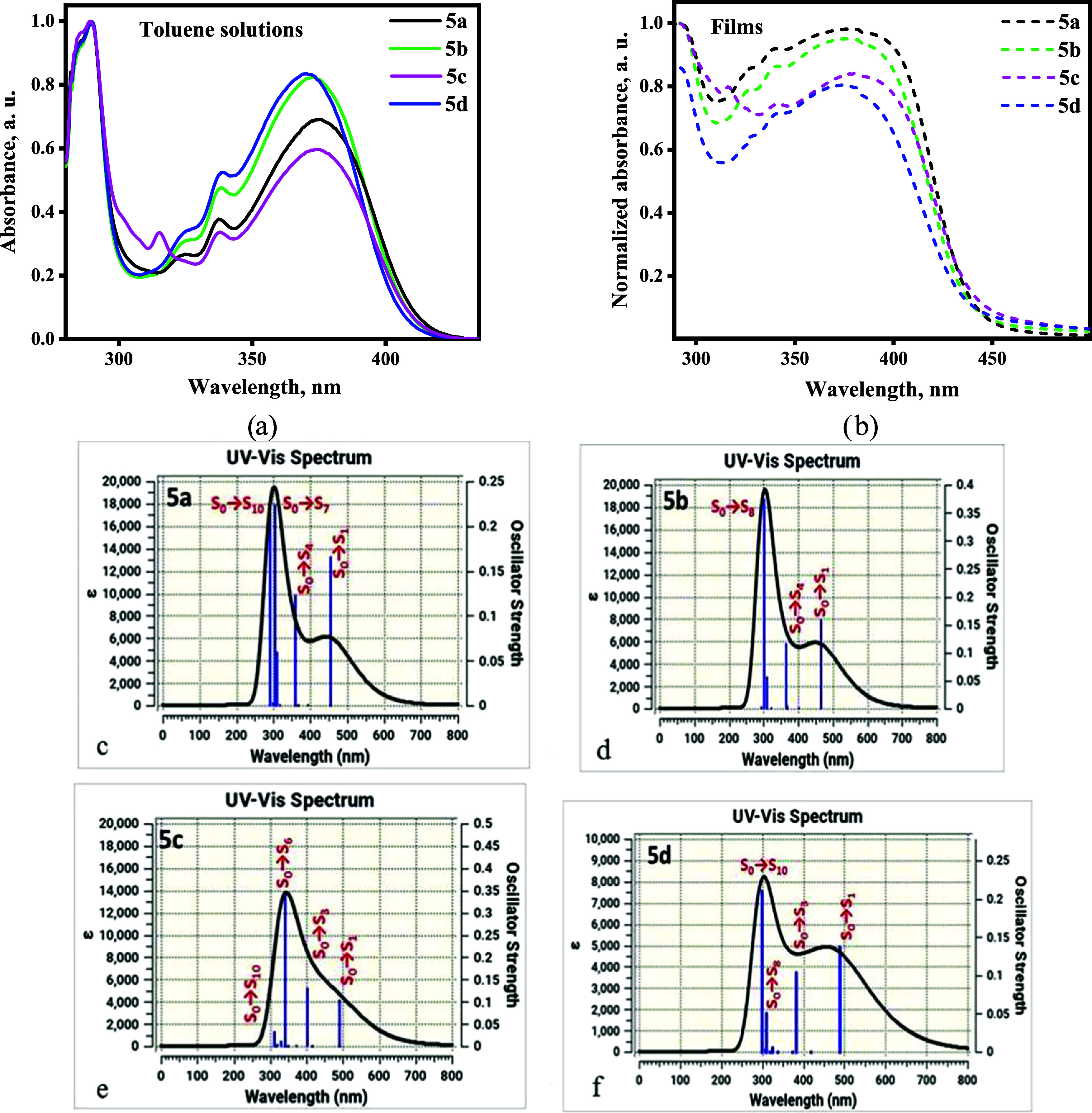
UV/vis spectra of dilute toluene solutions (a) and neat films (b) and theoretical UV/vis spectra (c–f) of compounds **5a**–**5d**, simulated with Gaussian ’16 software.

UV/vis spectra of the toluene solutions and neat films of the triazole derivatives (**5a**–**d**) are presented in Figure 4a,b. The studied compounds absorbed electromagnetic radiation of up to 420 nm. Similar shapes and widths of the absorption spectra of the toluene solutions of the compounds were observed. They all demonstrated well-defined maxima at 290, 337, and 375 nm. These maxima can be attributed to the overlapping of the locally excited (LE) transitions of the donor and acceptor as well as the charge-transfer (CT) transition between the donor and acceptor, as discussed in more detail below. For all four compounds, the absorption spectra of solid films were slightly red-shifted in comparison with that of the toluene solutions. These shifts could be explained by enchanced intermolecular interactions in the solid state.

To determine the origin of the lowest energy bands (LEBs) observed in the UV/vis spectra, quantum chemical calculations were performed for compounds **5a**–**d** (Figure 4b). The LEB of compound **5a** resulted from the overlapping of transitions from the S_0_ ground state to the S_1_ and S_4_ excited states (H → L; H → L + 1). The S_0_ → S_1_ and S_0_ → S_4_ transitions are mainly of the ICT characteristics. Charge transfer occurred from the carbazole-donating unit to the triazole-accepting moiety (Figures 4b and S21, Table S2). The LEB of compound **5b** also resulted from the overlapping of the S_0_ → S_1_ (H → L) and S_0_ → S_4_ (H-3 → L; H → L + 1) transitions from the carbazole unit to the triazole moiety (Figures 4b and S22, Table S3). The LEB of compound **5c** resulted from the overlapping of S_0_ → S_1_ and S_0_ → S_3_ (H → L; H → L + 1) transitions, which also originated from charge transfer (Figures 4b and S23, Table S4). The LEB of compound **5d** also contributed to S_0_ → S_1_ (H → L) and S_0_ → S_4_ (H-3 → L; H → L + 1) electronic transitions (Figures 4b and S24, Table S5). It can be therefore stated that the origin of the LEBs of the neat films and toluene solutions of **5a**–**5d** with maxima at ca. 375 nm (Figure 4b) is intramolecular charge transfer.

Additionally, theoretical simulations of the UV/vis spectra also revealed electronic transitions toward excited states with higher energies. Figure 4b shows a considerable impact of transitions S_0_ → S_7_ and S_0_ → S_10_ for compound **5a**, S_0_ → S_8_ for compound **5b**, S_0_ → S_6_ for **5c**, and S_0_ → S_10_ for compound **5d**. These transitions resulted from the overlapping of intramolecular charge transfer and local excitation of the triazole moiety (Figures S4–S7). In our previous work,^29^ we described similar derivatives of benzoyl-1*H*-1,2,3-triazole that have donor carbazole fragments attached at the *ortho*- and *meta*-positions. In that case, LEBs resulted mainly from the *n*–π* transitions of the triazole moiety.

**Figure 5:**
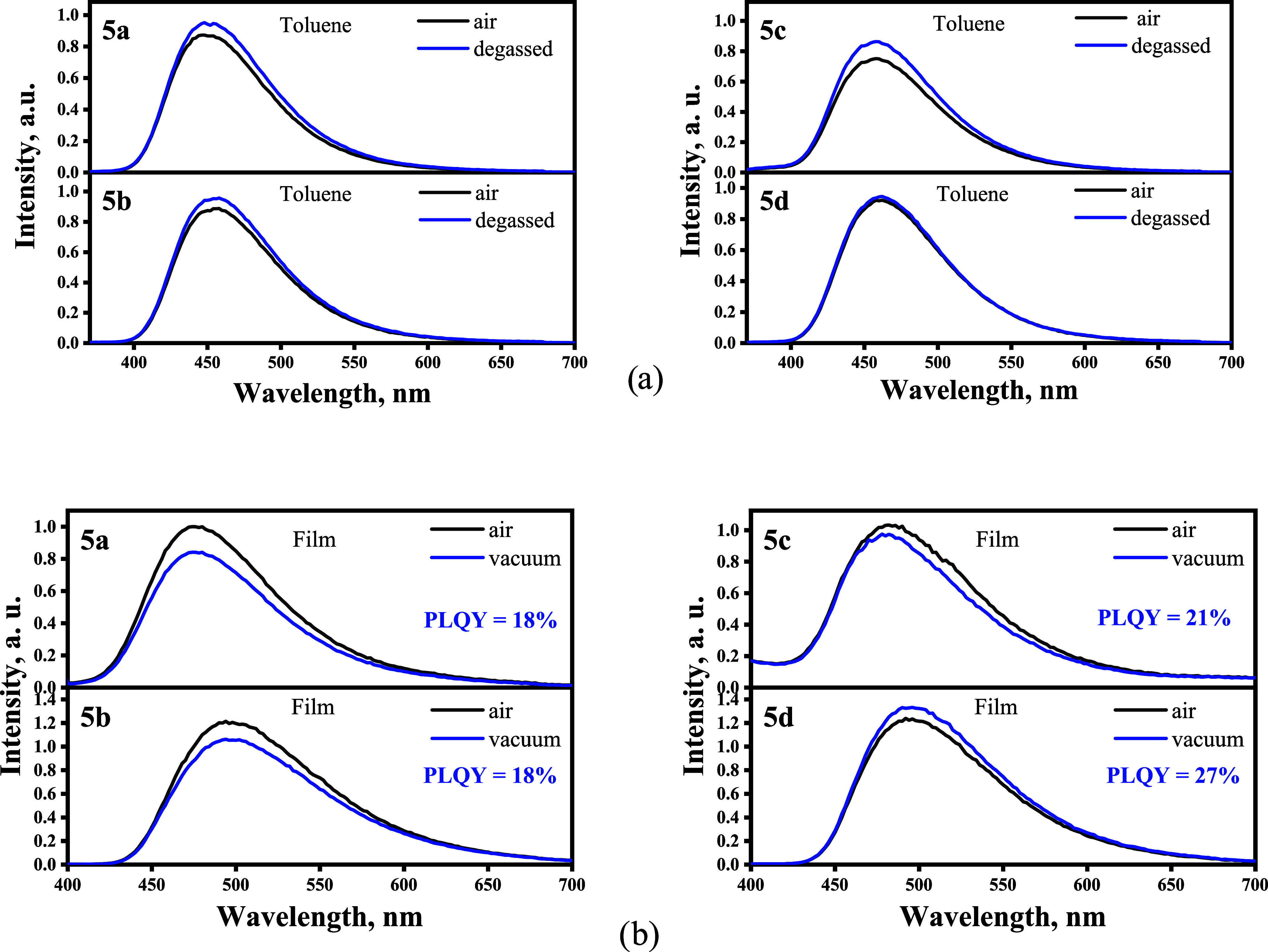
Photoluminescence spectra of degassed and air-equilibrated toluene solutions (a) and of solid films (b) of compounds **5d**–**5a** recorded in air and vacuum.

The photoluminescence (PL) spectra of 10^–5^ M toluene solutions of compounds **5a**–**5d** exhibited unstructured bands with well-defined intensity maxima at ca. 460 nm (Table 2, Figure 5a). All of the emission spectra were found to be Gaussian-shaped, which was indicative of charge transfer from donor to acceptor moieties^33^ and conformed with the results of computational studies. The emission maxima of the investigated compounds were found to be blue-shifted in comparison to those of our previously reported derivatives of benzoyl-1*H*-1,2,3-triazole (λ_max PL_ = 480 nm).^29^ This observation can be attributed to the effect of different substitution patterns. The carbazole fragment acts as a weaker electron donor when the phenyl linker is attached to the *para-*position in comparison to the *meta*- and *ortho-*linkages^34,35^ The position of the emission maxima of the toluene solutions of the investigated compounds correlated with the acceptor strength. Compound **5d** with a (*m-*trifluoromethyl)-phenyl substituent at the triazolyl-acceptor part exhibited the most red-shifted emission maximum at 460 nm, while compound **5a** with the (*m-*methyl)-phenyl fragment exhibited an emission maximum at 450 nm. No increase of PL intensity was observed after deoxygenation of the solutions. Figure S25 shows the PL decay curves of toluene solutions of compounds **5a**–**5d**. These were adequately represented by a single-exponential fit. No evidence of delayed fluorescence was observed. This observation together with the absence of a PL intensity increase after the removal of oxygen evidenced that the contribution of triplet excited states in the emission was rather negligible.

**Table 2:** Photophysical Characteristics of Compounds **5a**–**5d**

	**5a**	**5b**	**5c**	**5d**
λ_em_^a^ (nm)	450	455	457	460
λ_em_^b^ (nm)	474	492	484	488
Λ_abs_^c^ (nm)	289, 337, 375	289, 337, 375	289, 337, 372	289, 338, 370
Φ_F_^d^ (%)	18	18	21	27
τ_1_^air^	3.63	4.09	3.13	6.03
τ_2_^air^		15.10	9.15	
χ^2 air^	0.68	0.754	0.979	1.076
τ_1_^vacuum^	3.21	4.03	2.21	6.04
τ_2_^vacuum^		15.71	8.06	
χ^2^ vacuum	1.02	0.839	0.989	1.108
E_S1_ (eV)	3.90	3.05	3.06	3.05
E_T1_ (eV)	3.02	2.92	2.87	3.00
ΔE_ST_ (eV)	0.88	0.13	0.19	0.05

a Wavelengths of the emission maxima of 10^–5^ toluene solutions.

b Wavelengths of the emission maxima of the solid films.

c Wavelengths of the absorption maxima of 10^–5^ toluene solutions.

d Fluorescence quantum yields of solid samples were determined by a calibrated integrating sphere, τ_1_, τ_2_-photoluminescence lifetimes, χ^2^-double exponential function, E_S1_-energies of the first singlet excited states, E_T1_-energies of the first triplet excited states, and ΔE_ST_-singlet–triplet energy splitting.

The PL maxima of the solid films of compounds **5a**–**5d** (Figure 5b) were found to be bathochromically shifted with respect to those of toluene solutions. As long as the distance between molecules is considerably shortened in the solid state compared to dilute solutions, the redshifts can be attributed to intermolecular interactions. The most noticeable red shift of 37 nm was observed for compound **5b**. The PL quantum yields (PLQY, Φ_F_) of the solid films of compounds **5a** and **5c** were found to be 18%. Compound **5d** showed the highest PLQY of 27%. The PL lifetimes of the films were found to be longer than those of the toluene solutions (Figure S26). The films of compounds **5a** and **5c** exhibited single-exponential PL decays. For the solid films of compounds **5b** and **5d**, it was possible to fit the decay curves using a biexponential model. The longer relaxation pathway with τ_1_ = 4.09 ns and τ_2_ = 5.10 ns was observed for compound **5b**. After the removal of oxygen, the PL intensity of the films increased to a greater extent than in the case of the corresponding toluene solutions. Thus, for the solid films of the investigated triazole derivatives, the contribution of triplet excited states to emission was more pronounced than for the solutions.

**Figure 6:**
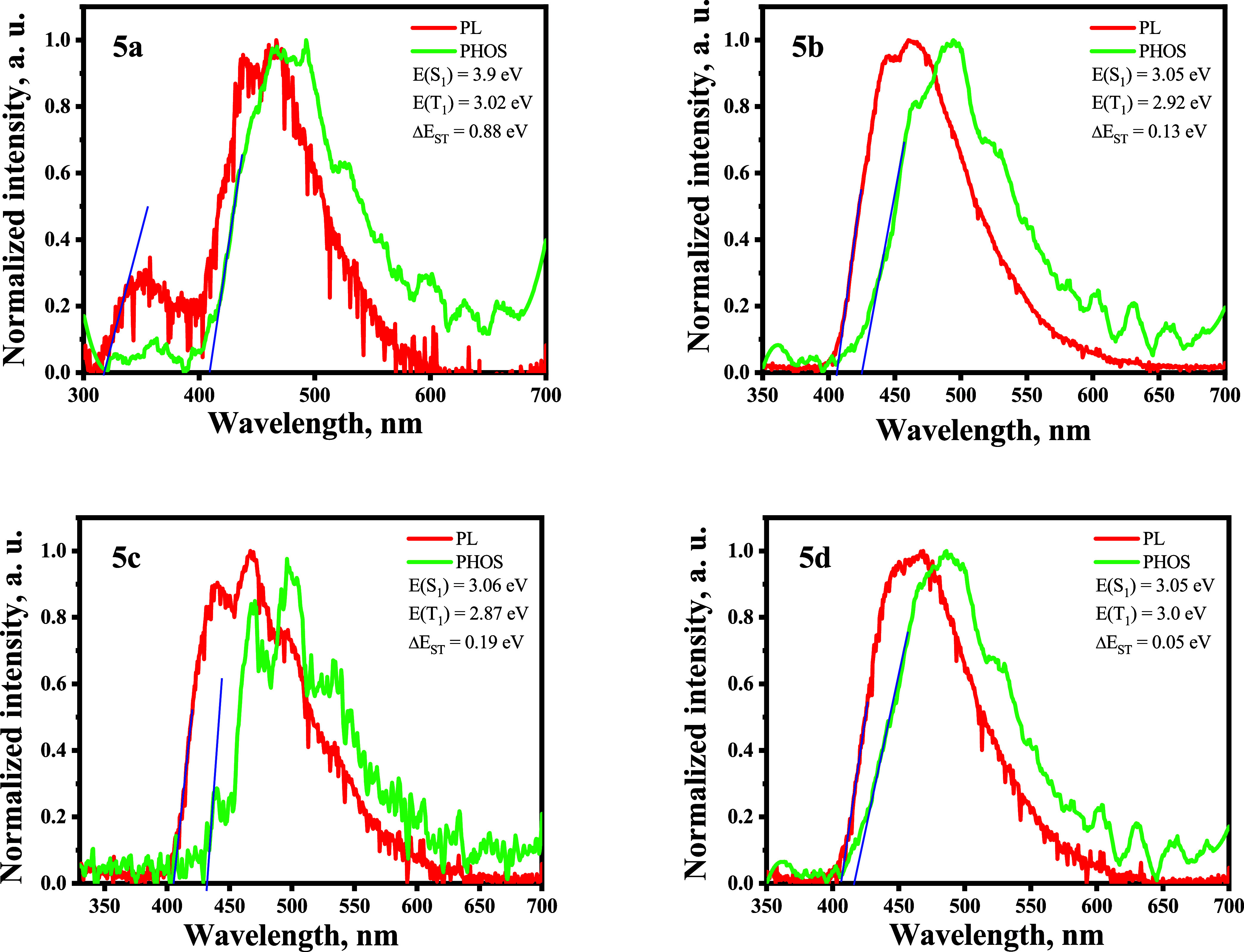
Photoluminescence and phosphorescence spectra of THF solutions of compounds **5a**–**5d** recorded at 77 K. Phosphorescence spectra were recorded using a delay of 50 ms after excitation. The S_1_ and T_1_ energy levels were calculated from the corresponding onsets, as shown by the blue lines.

In order to estimate the energies of the first singlet (S_1_) and first triplet (T_1_) excited states as well as singlet–triplet energy splitting (Δ*E*_ST_), the fluorescence and phosphorescence spectra of the THF solutions of compounds **5a**–**5d** were recorded at 77 K (Figure 6). The S_1_ energy values were estimated from the high-energy onsets of the fluorescence spectra. They were found to be in the range of 3.05–3.9 eV. The T_1_ values estimated from the onset of the phosphorescence spectra were in the range of 2.87–3.02 eV. Compounds **5a** and **5c** were characterized by a relatively wide energy gap between the energy levels S_1_ and T_1_. In contrast, compounds **5b** and **5d** possessed rather low Δ*E*_ST_ of 0.13 and 0.05 eV, respectively. Such small Δ*E*_ST_ values ensure thermally activated delayed fluorescence (TADF). They promote efficient singlet–triplet upconversion from not-radiative T_1_ excitons to radiative S_1_ excitons.

### Thermal Analysis

**Figure 7:**
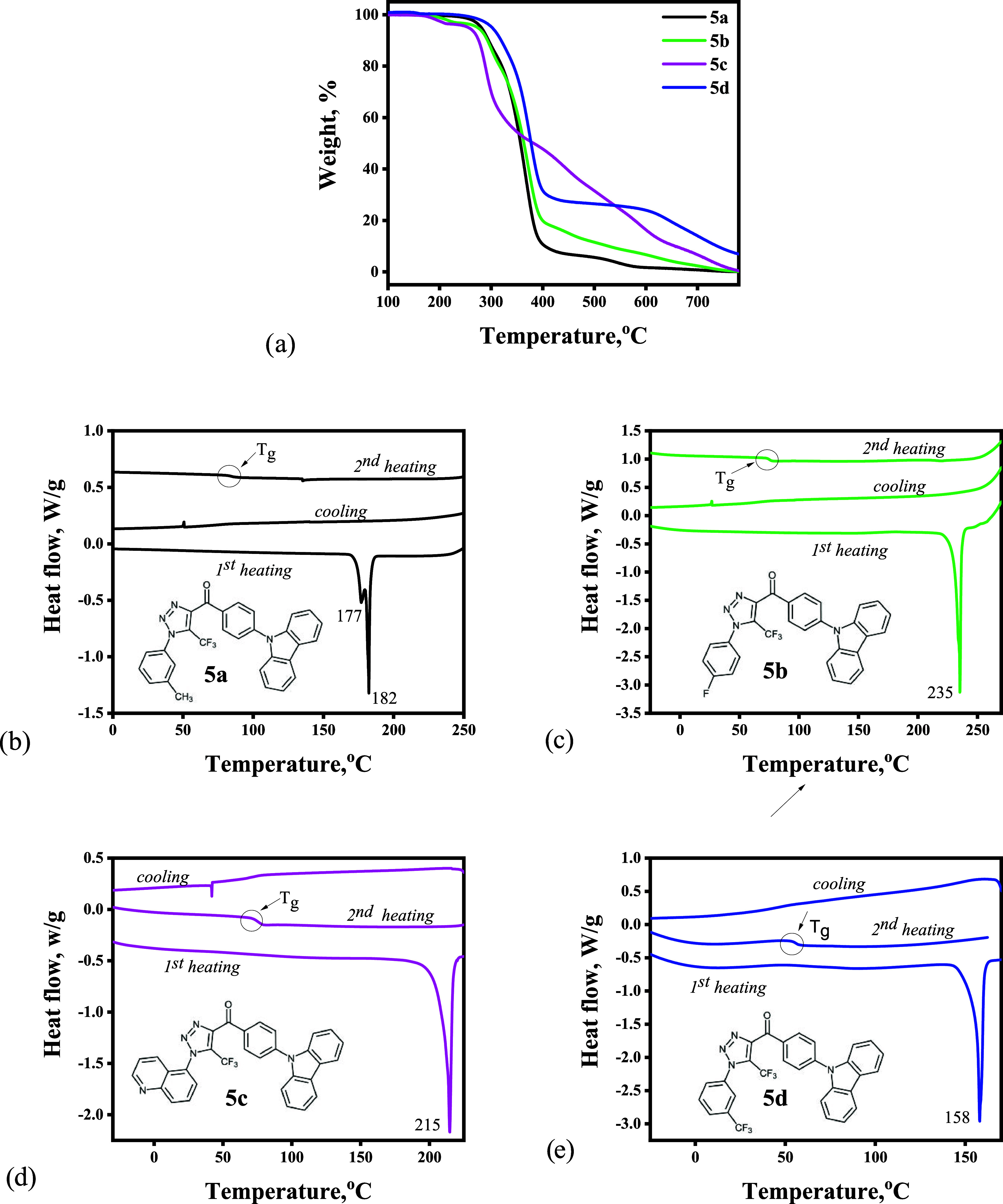
TGA (a) and DSC (b–e) curves of compounds **5a**–**d**.

The thermal properties of compounds **5a**–**d** (Figure 7) were investigated by employing thermogravimetric analysis (TGA) and differential scanning calorimetry (DSC) under an inert atmosphere. A summary of their thermal characteristics is given in Table 3. The thermal stability of the investigated compounds was strongly influenced by the substituents on the triazole ring. Trifluoromethyl substituents are known to improve the thermal stability of organic semiconductors.^36^ In the case of compound **5d**, the incorporation of one extra CF_3_ group increased the temperature of the onset of thermal degradation to 319 °C. For compounds **5a** and **5b**, 10% mass loss occurred at 295 and 294 °C, respectively. For quinoline-containing compound **5c**, a 10% mass loss occurred at 276 °C.

**Table 3:** Thermal Characteristics of Compounds **5a**–**d^a^**

compound	*T*_dec-10%_, °C	*T*_g_, °C	*T*_m_, °C
**5a**	295	84	177, 182
**5b**	294	75	235
**5c**	276	75	215
**5d**	319	54	158

a *T*_dec-10%_—temperature of 10% weight loss; *T*_g_— temperature of glass transition; *T*_m_—temperature of melting point.

All of the compounds were obtained as yellow crystals. The DSC measurements proved their crystalline nature. During the first heating scan, compound **5b** exhibited the highest melting point at 235 °C. Compound **5a** exhibited two closely located endothermic melting signals at 177 and 182 °C. This observation can be explained by the presence of two different types of crystals in the sample (polymorphism).^37^ Quinoline-containing compound (**5c**) showed a melting signal at 215 °C. No crystallization peaks were observed during the cooling process for any compound. In the second heating scan, compounds **5b** and **5c** showed identical glass transition temperatures at 75 °C. For compound **5d**, the transformation into a glassy state occurred at 54 °C. Compound **5a**, which contains a 3-methylphenyl moiety, exhibited a considerably higher glass transition temperature of 84 °C.

### Electroluminescence

**Figure 8:**
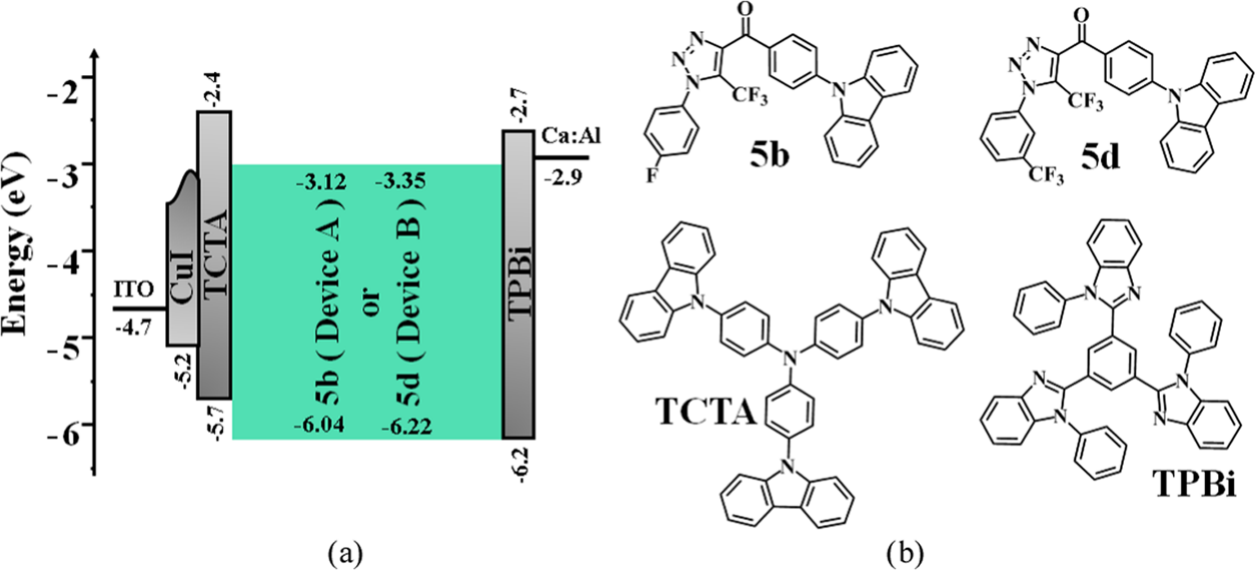
Equilibrium energy diagrams (a) of devices A and B, and molecular structures (b) of the functional materials.

Taking into account the smallest Δ*E*_ST_ values (Table 2), the EL properties of compounds **5b** and **5d** were studied by constructing an OLED with an ITO/CuI (2 nm)/TCTA (20 nm) emitting layer (EML) (70 nm)/ TPBi (20 nm)/Ca/Al (Figure 8a). Such a device structure is very similar to that of the single-layer CzDBA-based OLEDs mentioned in the introduction.^2^ Commercial materials copper(I) iodide (CuI), tris(4-carbazoyl-9-ylphenyl)amine (TCTA), and 2,2′,2″-(1,3,5-benzinetriyl)-tris(1-phenyl-1H-benzimi-dazole) (TPBi) were used as functional materials in the fabrication of OLEDs (Figure 8b). For hole injection, a TCTA layer, in combination with CuI,^38^ was used.^39^ The TPBi layer was used as the electron-transporting layer. Electron injection was done from the Ca cathode with a low work function of 2.9 eV (Figure 8a). In addition, blocking of triplets within the EML can be expected due to the high triplet levels of TCTA (2.8 eV^40^) and TPBi (2.7 eV^41^).

**Figure 9:**
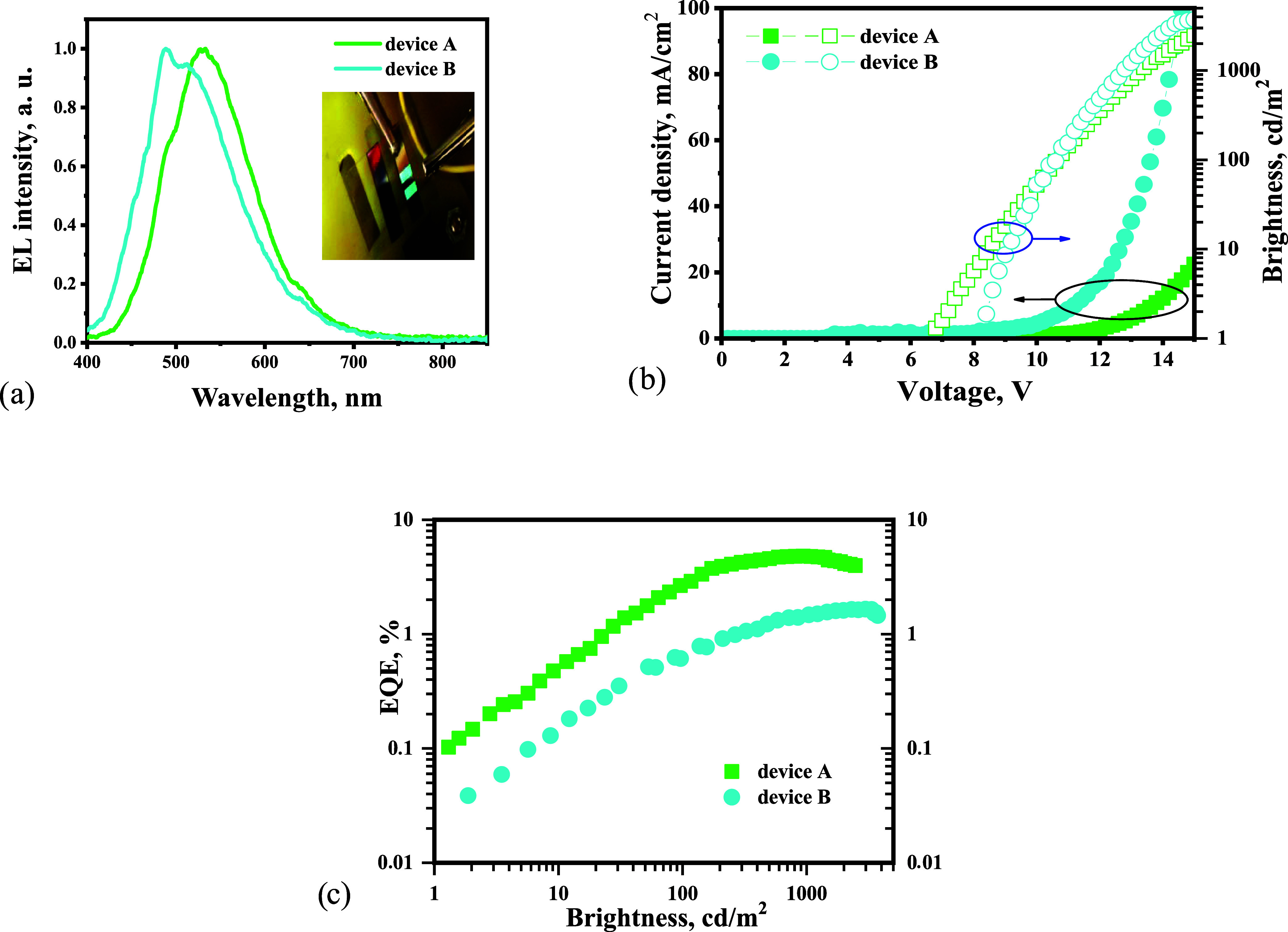
EL spectra (a), current density and brightness versus external voltage plots (b), and EQE as a function of current density (c) of devices A and B.

The EL spectra of devices A and B peaked at 531 and 490 nm, respectively, confirming emission from **5b** and **5d** (Figure 9a). The bluish-green and greenish-blue EL colors of devices A and B with CIE coordinates (0.34, 0.46) and (0.28, 0.38), respectively, were obtained (Figure 8a, Table 4). The electroluminescence (∼1 cd/m^2^) of devices A and B was observed at relatively high turn-on voltages (*V*_on_) of 6.8 and 8.2 V, respectively (Figure 9b, Table 4). Such *V*_on_ values can be attributed to the injection properties of the fabricated devices, which did not contain many functional layers. It is evident that further chemical modification of emitters is needed to achieve state-of-the-art parameters of blue single-layer OLEDs.

**Table 4:** Output EL Parameters of Devices A and B

device	EML	*V*_on_,^a^ (V)	max. brightness, cd/m^2,^^b^	CE_max_, cd/A^c^	EQE_max_, %^d^	λ_EL_, nm^e^	CIE (*x*; *y*)^f^
A	**5b**	6.8	2465	13.2	4.6	531	(0.34, 0.46)
B	**5d**	8.2	3742	3.7	1.6	490	(0.28, 0.38)

a Turn-on voltage.

b Maximum brightness.

c Maximum current efficiency.

d Maximum external quantum efficiency.

e Wavelengths of the electroluminescence maxima.

f Commision Internationale l’Eclaraige color coordinates.

Nevertheless, the brightness of devices A and B exceeded 1000 cd/m^2^ and reached a maximum value of 3742 cd/m^2^ in the case of device B (Figure 9b, Table 4). Device A showed a higher maximum EQE of 4.6% than that of device B (1.6%) (Figure 9c). These values were obtained at a high brightness of ca. 1000 cd/m^2^. This apparently can be explained by the hole–electron disbalance at low current densities. In addition, these values are not in agreement with the trend of the PLQY values of the films of **5b** (18%) and **5d** (27%) (Table 2). Thus, the efficiency of the simplified OLED A and B was more sensitive to the charge-injecting and charge-transporting properties than the PLQY of the EMLs of **5b** and **5d**. It should be noted that compounds **5b** and **5d** were characterized by triplet harvesting via TADF. This claim is supported by the relatively high EQE of 4.6% for device A containing emitter **5b**, which is characterized by a PLQY of 18%.

## Conclusions

Four D–A-type derivatives of triazoles decorated with different substituents were designed, synthesized, and characterized. The effect of modification of the acceptor site on the properties of the compounds was studied. The compounds containing 4-fluorophenyl and (3-trifluoromethyl)-phenyl fragments have demonstrated promising properties. They formed molecular glasses with glass transition temperatures of 75 and 54 °C, respectively. A 10% weight loss was observed at 294 and 319 °C, respectively. The bluish-green emission of the compounds is assigned to the charge transfer from the carbazole donor moiety to the triazole acceptor. The origin of emission was thermally activated delayed fluorescence resulting from fast singlet–triplet upconversion. It is demonstrated that the introduction of fluorine atoms into the acceptor unit can result in the improvement of thermal properties, enhancement of photoluminescence quantum yield, and decrease of singlet–triplet energy splitting. These compounds were used for the preparation of light-emitting layers of host-free OLEDs with a simplified structure. A maximum external quantum efficiency of 4.6% was achieved for the device containing an emitting layer of 4-((9*H*-carbazol-9-yl)phenyl)(1-(*m*-tolyl)-5-(trifluoromethyl)-1*H*-1,2,3-triazol-4-yl)methanone. The fabricated OLED exhibited greenish-blue emission with electroluminescence characterized by Commission International de l′Eclairage (CIE) coordinates (0.34, 0.46). This research indicates that by carefully engineering the donor–acceptor-type molecules based on the 1*H*-1,2,3-triazole core, it is possible to obtain emitters for efficient OLEDs, even with a very simple structure.

## Experimental Section

### Instrumentation

^1^H and ^13^C NMR spectra were recorded on Varian Unity Plus 400 (400 and 101 MHz, respectively) and Bruker Avance 500 (500 and 126 MHz, respectively) spectrometers in DMSO-*d*_6_ and CDCl_3_-*d*_6_ solutions, respectively, using TMS or the residual peaks of the solvent (2.50 ppm for ^1^H nuclei and 39.5 ppm for ^13^C nuclei) as internal references. Mass spectral analyses were performed using an Agilent 1100 series LC/MSD in the API-ES/APCI mode (200 eV). Elemental analysis was performed using a Carlo Erba 1106 instrument. The melting points were determined on a Mel-Temp melting point apparatus. IR spectra were recorded on a Bruker VERTEX 70 Fourier-transform infrared (FT-IR) spectrometer. X-ray single-crystal diffraction was performed on a diffractometer with a CCD detector using a Cu Kα radiation source (λ = 1.5418 Å).

Differential scanning calorimetry (DSC) measurements were performed by using a TA Instruments Q2000 instrument. The samples were examined under a nitrogen atmosphere at a heating rate of 10 °C/min. Thermogravimetric analysis (TGA) was performed on a TA Instrument Q50 instrument. The heating rate was 20 °C/min under a nitrogen atmosphere.

The ground-state geometries were optimized by using the B3LYP (Becke three parameters hybrid functional with the Lee–Yang–Perdew correlation) functional at the 6-31G(d,p) level in vacuum with the Gaussian program. UV–vis spectra was generated by using the B3LYP (Becke three parameters hybrid functional with the Lee–Yang–Perdew correlation) functional at the 6-31G(d,p) level in a vacuum with the Gaussian program.

Cyclic voltammetry measurements were performed by using a platinum working electrode (a disk with a diameter of 2 mm) in a three-electrode cell with an Autolab-type potentiostat–galvanostat. The measurements were carried out for solutions in dry dichloromethane containing 0.1 M tetrabutylammonium hexafluorophosphate at 25 °C, with a scan rate of 50 mV/s and a sample concentration of 10^–3^ M. The potentials were measured against silver as a quasi-reference electrode. Platinum wire was used as the counter electrode. The potentials were calibrated with a standard ferrocene/ferrocenium (Fc/Fc^+^) redox system.

Thin solid films for the measurement of UV/vis and PL spectra were prepared by drop-casting 2 mg/mL toluene solutions of the compounds on precleaned quartz substrates. The UV/vis spectra of the solutions and thin films of the compounds were recorded by a PerkinElmer UV/vis Spectrometer Lambda 25.

An Edinburgh Instruments FLS980 spectrophotometer and a PicoQuant LDH-D-C-375 laser with an excitation wavelength of 374 nm were used to record the photoluminescence spectra of the solutions and thin films, and the corresponding photoluminescence decays. The phosphorescence spectra of the THF solutions were recorded at 77 K with a delay time after excitation (330 nm) exceeding 50 ms.

### Materials

#### Synthesis of (Bromoaryl)-(1-aryl-5-(trifluoromethyl)-1*H*-1,2,3-triazol-4-yl)methanones **3** (General Procedure)

A mixture of the corresponding 1-(bromoaryl)-4,4,4-trifluorobutane-1,3-dione **2** (3 mmol), the appropriate aryl azide **1** (3 mmol), and triethylamine (1.30 mL, 9 mmol) was heated at 70–75 °C for 5 h. After cooling to room temperature, the formed solid was mixed with isopropanol, filtered, and dried in air to give the target triazole **3**.

##### 4-(Bromophenyl)-(1-(*m*-tolyl)-5-(trifluoromethyl)-1*H*-1,2,3-triazol-4-yl)methanone **3a**

was obtained as a white solid: yield, 79%; m.p., 111–112 °C. ^1^H NMR (500 MHz, DMSO-*d*_6_): δ 8.04 (d, *J* = 7.9 Hz, 2H, H^ArBr^-2,6), 7.86 (d, *J* = 7.9 Hz, 2H, H^ArBr^-3,5), 7.57–7.50 (m, 4H, H^Ar^), 2.42 (s, 3H, CH_3_). ^13^C NMR (126 MHz, DMSO-*d*_6_): δ 184.54 (CO), 144.57 (C^Triazole^-4), 139.56 (C^Tol^-3), 139.28 (C^Tol^-1), 135.02 (C^ArBr^-1), 132.28 (2xCH^ArBr^-2,6), 132.06 (CH^Tol^-4), 132.01 (2xCH ^ArBr^-3,5), 129.41 (CH^Tol^-4), 129.21 (q, ^2^*J*_C–F_ = 41.5 Hz, C^Triazole^-5), 128.97 (C^ArBr^-4), 126.51 (CH^Tol^-2), 123.27 (CH^Tol^-6), 118.52 (q, ^1^*J*_C–F_ = 270.5 Hz, CF_3_), 20.68 (CH_3_). MS (*m*/*z*): 410, 412 (M^+^ + 1). Anal. calcd for C_17_H_11_BrF_3_N_3_O (410,1942): C, 49.78; H, 2.70; N, 10.24; found: C, 49.70; H, 2.93; N, 10.15.

##### (4-Bromophenyl)(1-(4-fluorophenyl)-5-(trifluoromethyl)-1*H*-1,2,3-triazol-4-yl)methanone **3b**

was obtained as a white solid: yield, 89%; m.p., 136–137 °C. ^1^H NMR (500 MHz, DMSO-*d*_6_): δ 8.05 (d, *J* = 7.9 Hz, 2H), 7.90–7.80 (m, 4H), 7.53 (t, *J* = 8.1 Hz, 2H, H^ArF^-3,5). ^13^C NMR (126 MHz, DMSO-*d*_6_): δ 184.44 (CO), 163.34 (d, ^1^*J*_C–F_ = 249.5 Hz, C^ArF^-4) 144.53 (C^Triazole^-4), 134.61 (C^ArBr^-1), 132.26 (2xCH^ArBr^-2,6), 132.00 (2xCH^ArBr^-3,5), 131.39 (d, ^4^*J*_C–F_ = 2.4 Hz, C^ArF^-1), 129.21 (d, ^2^*J*_C–F_ = 41.2 Hz, C^Triazole^-5), 128.98 (C^ArBr^-4), 128.92 (d, ^3^*J*_C–F_ = 9.5 Hz, 2xCH^ArF^-2,6) 118.83 (d, ^1^*J*_C–F_ = 270.4 Hz, CF_3_), 116.71 (d, ^2^*J*_C–F_ = 23.6 Hz, 2xCH^ArF^-3,5). MS (*m*/*z*): 414, 416 (M^+^ + 1). Anal. calcd for C_16_H_8_BrF_4_N_3_O (414,1576): C, 46.40; H, 1.95; N, 10.15; found: C, 46.53; H, 1.88; N, 10.14.

##### (4-Bromophenyl)(1-(quinolin-5-yl)-5-(trifluoromethyl)-1*H*-1,2,3-triazol-4-yl)methanone **3c**

was obtained as a white solid: yield, 88%; m.p., 187–188 °C. ^1^H NMR (500 MHz, DMSO-*d*_6_) δ 9.10 (d, *J* = 1.1 Hz, 1H, H^Quin^-2), 8.42 (d, *J* = 7.2 Hz, 1H, H^Quin^-4), 8.18 (d, *J* = 6.0 Hz, 2H, H^Ar^-2,6), 8.12 (d, *J* = 5.9 Hz, 1H, H^Quin^-8), 8.08–7.99 (m, 2H, H^Quin^-6,7), 7.91 (d, *J* = 6.4 Hz, 2H, H^ArBr^-3,5), 7.70 (br.s, 1H, H^Quin^-3). ^13^C NMR (126 MHz, DMSO-*d*_6_) δ 184.85 (CO), 152.70 (CH^Quin^-2), 147.84 (C^Quin^-8a), 145.23 (C^Triazole^-4), 135.17 (C^ArBr^-1), 133.52 (CH^Quin^-4), 132.99 (2xCH^ArBr^-2,6), 132.43 (2xCH^ArBr^-3,5), 131.34 (CH^Quin^-8), 131.08 (CH^Quin^-6), 129.44 (d, ^2^*J*_C–F_ = 41.4 Hz, C^Triazole^-5), 129.40 (C^ArBr^-4), 129.34 (CH^Quin^-7), 126.98 (CH^Quin^-4), 124.87 (C^Quin^-4a), 124.10 (CH^Quin^-3), 119.31 (q, ^1^*J*_C–F_ = 270.6 Hz, CF_3_). MS (*m*/*z*): 447, 449 (M^+^ + 1). Anal. calcd for C_19_H_10_BrF_3_N_4_O (445,9990): C, 51.03; H, 2.25; N, 12.53; found: C, 51.11; H, 2.27; N, 12.49.

##### 4-Bromophenyl(5-(trifluoromethyl)-1-(3-(trifluoromethyl)phenyl)-1*H*-1,2,3-triazol-4-yl)methanone **3d**

was obtained as a white solid: yield, 79%; m.p., 170–171 °C. ^1^H NMR (500 MHz, DMSO-*d*_6_) δ 8.29 (s, 1H, H^Ar^–2), 8.18–8.11 (m, 2H, H^Ar^–4,6), 8.07 (d, *J* = 7.2 Hz, 2H, H^ArBr^–2,6), 7.97 (t, *J* = 7.0 Hz, 1H, H^Ar^–5), 7.89 (d, *J* = 6.8 Hz, 2H, H^ArBr^–3,5). ^13^C NMR (101 MHz, DMSO-*d*_6_) δ 184.94 (CO), 145.04 (C^Triazole^–4), 136.18 (C^Ar^–1), 135.07 (C^ArBr^–1), 132.74 (2xCH^ArBr^–2,6), 132.55 (2xCH^ArBr^–3,5), 131.66 (CH^ArCF^–6), 131.20 (CH^ArCF^–5), 130.73 (q, ^2^*J*_C–F_ = 32.8 Hz, C^ArCF^–3), 129.84 (q, ^2^*J*_C–F_ = 41.6 Hz, C^Triazole^–5), 129.59 (C^ArBr^–4), 128.85 (q, ^3^*J*_C–F_ = 3.2 Hz, CH^ArCF^–4), 124.27 (q, ^3^*J*_C–F_ = 2.9 Hz, CH^ArCF^–2), 123.79 (q, ^1^*J*_C–F_ = 272.8 Hz, CF_3_), 119.26 (q, ^1^*J*_C–F_ = 271.0 Hz, CF_3_^Triazole^). MS (*m*/*z*): 464, 466 (M^+^ + 1); Anal. calcd for C_17_H_8_BrF_6_N_3_O (462, 9755): C, 43.99; H, 1.74; N, 9.05; found: C, 43.90; H, 1.93; N, 9.00.

#### Synthesis of (9*H*-carbazol-9-yl-aryl)-(1-aryl-5-(trifluoromethyl)-1*H*-1,2,3-triazol-4-yl)methanones **5** (General Procedure)

In a Schlenk flask (25 mL), bromo-containing triazoles **3** (0.671 mmol), carbazole **4** (0.2 g, 1.197 mmol), potassium carbonate (0.2 g, 1.45 mmol), copper powder (10 mg, 0.156 mmol), copper(I)chloride (10 mg, 0.100 mmol), 1,10-phenanthroline (20 mg, 0.12 mmol), and xylene (7.0 mL) were placed. The reactions were performed under gentle stirring and refluxing (oil bath, temperature ≈ 150 °C) for 36–48 h (TLC control). After completion of the reaction, the mixture was filtered through a Celite layer. All volatiles were evaporated in a vacuum. The residue was purified by silica gel column chromatography. The obtained compounds **5** were additionally recrystallized from appropriate solvents.

##### (4-(9*H*-carbazol-9-yl)phenyl)(1-(*m*-tolyl)-5-(trifluoromethyl)-1*H*-1,2,3-triazol-4-yl)methanone **5a**

was obtained as yellow crystals: yield, 41%; m.p., 147–149 °C; eluent for column chromatography, DCM/Hex (2:1); recrystallized from ethanol. ^1^H NMR (400 MHz, CDCl_3_) δ 8.52 (d, *J* = 8.5 Hz, 2H), 8.18 (d, *J* = 7.7 Hz, 2H), 7.84 (d, *J* = 8.5 Hz, 2H), 7.59 (d, *J* = 8.2 Hz, 2H), 7.53–7.46 (m, 4H), 7.43–7.34 (m, 4H), 2.53 (s, 3H). ^13^C NMR (101 MHz, CDCl_3_) δ 183.86 (C=O), 143.24 (C^Triazole^-4), 140.12 (2xC^Carbazole^-8a,9a), 140.08 (C^Ar^-4), 135.38 (C^Tol^-1), 134.11 (C^Tol^-3), 132.63 (2xCH^Ar^-2,6), 132.01 (CH^Tol^-4), 129.84 (q, ^2^*J*_C–F_ = 41.6 Hz, C^Triazole^-5), 129.38 (CH^Tol^-4), 126.36 (2xCH^Carbazole^-2,7), 126.30 (2xCH^Carbazole^-3,6), 126.18 (CH^Tol^-2), 124.02 (2xC^Carbazole^-4a,4b), 122.68 (CH^Tol^-6), 120.79 (2xCH^Carbazole^-4,5), 120.47 (2xCH^Ar^-3,5), 118.99 (q, ^1^*J*_C–F_ = 270.9 Hz, CF_3_^Triazole^), 109.91 (2xCH^Carbazole^-1,8), 21.29 (CH_3_). ^19^F NMR (376 MHz, CDCl_3_) δ −56.08 (CF_3_). MS (*m*/*z*): 497 (M^+^ + 1). IR ν_max_ (KBr) cm^–1^: 3080, 3054, 3018 (−C–H Ar), 2994, 2943, 2925 (−C–H Aliph.), 1678, 1562, (C=O); 1360, 1335, 1316 (−C–N−); 1198, 1124 (−CF_3_). Anal. calcd for C_29_H_19_F_3_N_4_O (496,4932): C, 70.16; H, 3.86; N, 11.28; found: C, 70.07; H, 3.81; N, 11.21.

##### (4-(9*H*-Carbazol-9-yl)phenyl)(1-(4-fluorophenyl)-5-(trifluoromethyl)-1*H*-1,2,3-triazol-4-yl)methanone **5b**

was obtained as bright yellow needles: yield, 67%; m.p., 150–151 °C; eluent for column chromatography, DCM/Hex (1:1); recrystallized from ethanol. ^1^H NMR (400 MHz, CDCl_3_) δ 8.52 (td, *J* = 8.8, 2.1 Hz, 1H), 8.18 (d, *J* = 7.7 Hz, 1H), 7.85 (td, *J* = 8.8, 1.9 Hz, 1H), 7.64–7.58 (m, 2H), 7.50–7.45 (m, 1H), 7.39–7.33 (m, 2H). ^13^C NMR (101 MHz, CDCl_3_) δ 183.65 (CO), 161.30 (d, ^1^*J*_C–F_ = 251.5 Hz, C^ArF^-4), 145.84 (C^Triazole^-4), 143.35 (C^Ar^-4), 140.10 (2xC^Carbazole^-8a,9a), 133.96 (C^ArF^-1), 132.62 (2xCH^Ar^-2,6), 128.37 (q, ^2^*J*_C–F_ = 41.6 Hz, C^Triazole^-5), 127.80 (d, ^3^*J*_C–F_ = 9.2 Hz, 2xCH^ArF^-2,6), 126.37 (2xCH^Carbazole^-2,7), 126.30 (2xCH^Carbazole^-3,6), 124.03 (2xC^Carbazole^-4a,4b), 120.83 (2xCH^Carbazole^-4,5), 120.49 (2xCH^Ar^-3,5), 118.91 (d, *J* = 298.7 Hz)116.91 (d, ^2^*J*_C–F_ = 23.5 Hz, 2xCH^ArF^-3.5), 109.89 (2xCH^Carbazole^-1,8). ^19^F NMR (376 MHz, CDCl_3_) δ −56.07 (CF_3_), −107.70 (C–F^Ar^). MS (*m*/*z*): 501 (M^+^ + 1). IR ν_max_ (KBr) cm^–1^: 3087, 3045 (−C–H Ar), 1678, 1595, (C=O); 1337, 1316, 1290 (−C–N−); 1187, 1157 (−CF_3_); 1102, 1083 (−C–F). Anal. calcd for C_16_H_8_BrF_4_N_3_O (414,16): C, 46.40; H, 1.95; N, 10.15; found: C, 46.47; H, 1.83; N, 10.21.

##### (4-(9*H*-Carbazol-9-yl)phenyl)(1-(quinolin-5-yl)-5-(trifluoromethyl)-1*H*-1,2,3-triazol-4-yl)methanone **5c**

was obtained as a yellow powder: yield, 63%; m.p., 202–204 °C; eluent for column chromatography, DCM; recrystallized from ethanol-DMF (9:1). ^1^H NMR (500 MHz, CDCl_3_) δ 9.02 (dd, *J* = 4.1, 1.5 Hz, 1H), 8.51 (td, *J* = 8.8, 2.1 Hz, 2H), 8.39 (d, *J* = 8.6 Hz, 1H), 8.10 (d, *J* = 7.6 Hz, 2H), 7.86 (dd, *J* = 8.6, 7.4 Hz, 1H), 7.79 (td, *J* = 8.8, 2.1 Hz, 2H), 7.70 (d, *J* = 7.4 Hz, 1H), 7.59 (d, *J* = 8.2 Hz, 1H), 7.52 (d, *J* = 8.2 Hz, 2H), 7.49 (dd, *J* = 8.6, 4.2 Hz, 1H), 7.39 (ddd, *J* = 8.3, 7.2, 1.2 Hz, 2H), 7.32–7.25 (m, 2H). ^13^C NMR (126 MHz, CDCl_3_) δ 183.53 (CO), 151.98 (CH^Quin^-2), 148.22 (C^Quin^-8a), 145.52 (C^Triazole^-4), 143.44 (C^Ar^-4), 140.09 (2xC^Carbazole^-8a,9a), 133.94 (C^Ar^-1), 133.76 (CH^Quin^-4), 132.71 (2xCH^Ar^-2,6), 132.08 (q, ^2^*J*_C–F_ = 42.0 Hz, C^Triazole^-5), 131.15 (C^Quin^-5), 129.97 (CH^Quin^-8), 128.30 (CH^Quin^-6), 126.42 (2xCH^Carbazole^-2,7), 126.35 (2xCH^Carbazole^-3,6), 125.78 (CH^Quin^-4), 124.91 (C^Quin^-4a), 124.07 (CH^Quin^-3), 123.28 (2xC^Carbazole^-4a,4b), 120.89 (2xCH^Carbazole^-4,5), 120.55 (2xCH^Ar^-3,5), 118.94 (q, ^1^*J*_C–F_ = 271.2 Hz, CF_3_), 109.92 (2xCH^Carbazole^-1,8). ^19^F NMR (471 MHz, CDCl_3_) δ −57.81 (CF_3_). MS (*m*/*z*): 534 (M^+^ + 1). IR ν_max_ (KBr) cm^–1^: 3069, 3055, 3023 (−C–H Ar), 1663, 1612, 1563 (C=O); 1338, 1327, 1289 (−C–N−); 1180, 1163, 1124 (−CF_3_). Anal. calcd for C_31_H_18_F_3_N_5_O (533,1463): C, 69.79; H, 3.40; N, 13.13; found: C, 69.71; H, 3.49; N, 13.17.

##### (4-(9*H*-Carbazol-9-yl)phenyl)(5-(trifluoromethyl)-1-(3-(trifluoromethyl)phenyl)-1*H*-1,2,3-triazol-4-yl)methanone **5d**

was obtained as light-yellow crystals: yield, 64%; m.p., 162–163 °C; eluent for column chromatography, DCM/Hex (1:1); recrystallized from ethanol. ^1^H NMR (400 MHz, CDCl_3_) δ 8.52 (td, *J* = 8.8, 2.0 Hz, 2H), 8.18 (d, *J* = 7.7 Hz, 2H), 8.00–7.96 (m, 1H), 7.94 (s, 1H), 7.86 (t, *J* = 1.8 Hz, 1H), 7.85–7.82 (m, 3H), 7.60 (d, *J* = 8.2 Hz, 2H), 7.50–7.46 (m, 2H), 7.39–7.35 (m, 2H). ^13^C NMR (101 MHz, CDCl_3_) δ 183.44 (C=O), 146.09 (C^Triazole^-4), 143.45 (C^Ar^-4), 140.08 (2xC^Carbazole^-8a,9a), 135.85 (C^ArCF^-1), 133.83 (C^Ar^-1), 132.64 (d, *J* = 33.9 Hz, C^ArCF^-3), 132.62 (2xCH^Ar^-2,6), 130.53 (CH^ArCF^-6), 129.84 (q, ^2^*J*_C–F_ = 41.6 Hz, C^Triazole^-5), 128.94 (CH^ArCF^-5), 128.16 (q, *J* = 3.5 Hz, CH^ArCF^-4), 126.39 (2xCH^Carbazole^-2,7), 126.32 (2xCH^Carbazole^-3,6), 124.05 (2xC^Carbazole^-4a,4b), 122.97 (q, *J* = 272.8 Hz, CF_3_^ArCF^), 122.98 (q, *J* = 3.2 Hz, CH^ArCF^-2), 120.86 (2xCH^Carbazole^-4,5), 120.50 (2xCH^Ar^-3,5), 118.99 (q, *J* = 270.9 Hz, CF_3_^Triazole^), 109.89 (2xCH^Carbazole^-1,8). ^19^F NMR (376 MHz, CDCl_3_) δ −55.84, −62.89. MS (*m*/*z*): 551 (M^+^ + 1). IR ν_max_ (KBr) cm^–1^: 3072, 3055, 3019 (−C–H Ar), 1668, 1609, 1570 (C=O); 1338, 1325, 1271 (−C–N−); 1174, 1159, 1120 (−CF_3_). Anal. calcd for C_29_H_16_F_6_N_4_O (550,4644): C, 63.28; H, 2.93; N, 10.18; found: C, 63.37; H, 2.84; N, 10.05.

## Supporting Information Available

1H NMR spectrum of compound **3a** in DMSO-*d*_6_ (Figure S1); ^13^C NMR spectrum of compound **3a** in DMSO-*d*_6_ (Figure S2); ^1^H NMR spectrum of compound **3b** in DMSO-*d*_6_ (Figure S3); ^13^C NMR spectrum of compound **3b** in DMSO-*d*_6_ (Figure S4); ^1^H NMR spectrum of compound **3c** in DMSO-*d*_6_ (Figure S5); ^13^C NMR spectrum of compound **3c** in DMSO-*d*_6_ (Figure S6); ^1^H NMR spectrum of compound **3d** in DMSO-*d*_6_ (Figure S7); ^13^C NMR spectrum of compound **3d** in DMSO-*d*_6_ (Figure S8); ^1^H NMR spectrum of compound **5a** in CDCl_3_ (Figure S9); ^13^C NMR spectrum of compound **5a** in CDCl_3_ (Figure S10); ^19^F NMR spectrum of compound **5a** in CDCl_3_ (Figure S11); ^1^H NMR spectrum of compound **5b** in CDCl_3_ (Figure S12); ^13^C NMR spectrum of compound **5b** in CDCl_3_ (Figure S13); ^19^F NMR spectrum of compound **5b** in CDCl_3_ (Figure S14); ^1^H NMR spectrum of compound **5c** in CDCl_3_ (Figure S15); ^13^C NMR spectrum of compound **5c** in CDCl_3_ (Figure S16); ^19^F NMR spectrum of compound **5c** in CDCl_3_ (Figure S17); ^1^H NMR spectrum of compound **5d** in CDCl_3_ (Figure S18); ^13^C NMR spectrum of compound **5d** in CDCl_3_ (Figure S19); ^19^F NMR spectrum of compound **5d** in CDCl_3_ (Figure S20); visualization of electronic transitions for compounds **5a**–**5d**, simulated with Gaussian’16 software (Figures S21–S24); photoluminescence decay curves of compounds **5a**–**5d** in toluene solutions, degassed, and air-equilibrated (Figure S25); photoluminescence decay curves of compounds **5a**–**5d** films before and after evacuation (Figure S26); crystal data and structure refinements of compound **5b** (Table 1); characterization of electronic transitions for compounds **5a**–**5d** (Tables S2–S5) (PDF)

## Footnotes

1 Cambridge Crystallographic Data Centre Identification Number CCDC 2248124.
