# Supplementary material for: Effects of Electron-Withdrawing Strengths of the Substituents on the Properties of 4-(Carbazolyl-R-benzoyl)-5-CF3-1H-1,2,3-triazole Derivatives as Blue Emitters for Doping-Free Electroluminescence Devices
Source: ACS Omega. 2024 Mar 12;9(12):14613–26. doi: 10.1021/acsomega.4c01077 (PMC10976381; doi:10.1021/acsomega.4c01077)
Supplement: Supplementary file 1 — ao4c01077_si_001.pdf [file ao4c01077_si_001.pdf]

Effects of electron-withdrawing strengths of the substituents on the properties of 4-(carbazolyl-R-benzoyl)-5-CF_3-1*H*-1,2,3-triazole derivatives as blue emitters for doping-free electroluminescent devices

Mariia Stanitska[1,2], Nazariy Pokhodylo[2], Roman Lytvyn[2], Ervinas Urbonas[1], Dmytro Volyniuk[1], Stepan Kutsiy[3], Khrystyna Ivaniuk[3], Vasyl Kinzhybalo[4], Pavlo Stakhira[3], Rasa Keruckiene[1], Mykola Obushak[2], Juozas Vidas Gražulevičius*[1]

[1]*Kaunas University of Technology, Baršausko st. 59, 51423 Kaunas, Lithuania. *email: juozas.grazulevicius@ktu.lt;*

[2]*Ivan Franko National University of Lviv, Kyryla i Mefodiya 6, Lviv, Ukraine,*

[3]*National University "Lviv Polytechnic", Stepan Bandera 12, Lviv, Ukraine*

[4]*Institute of Low Temperature and Structure Research, Okólna 2, Wrocław, Poland*

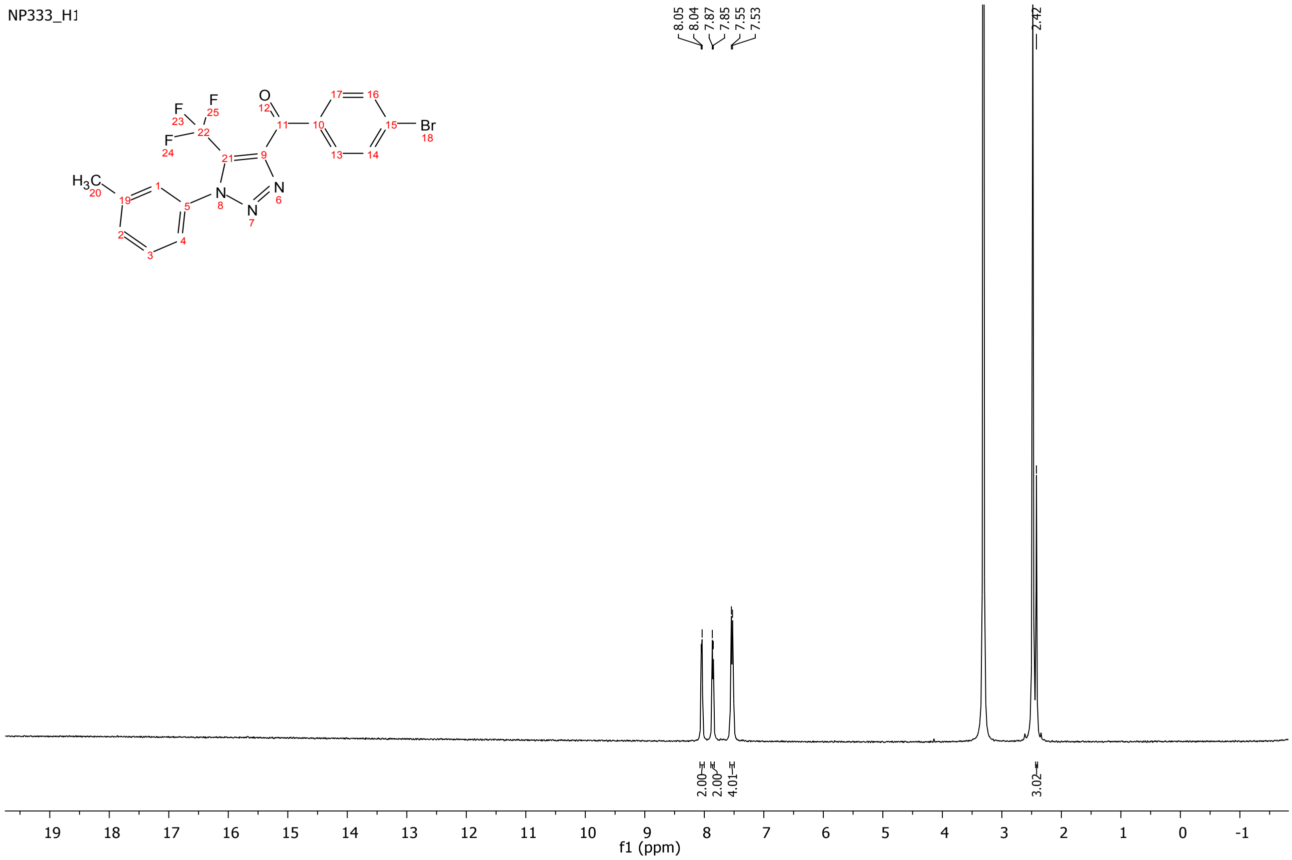

Figure S1. ^{1}H NMR (4-Bromophenyl)(1-(m-tolyl)-5-(trifluoromethyl)-1H-1,2,3-triazol-4-

yl)methanone **3a**.

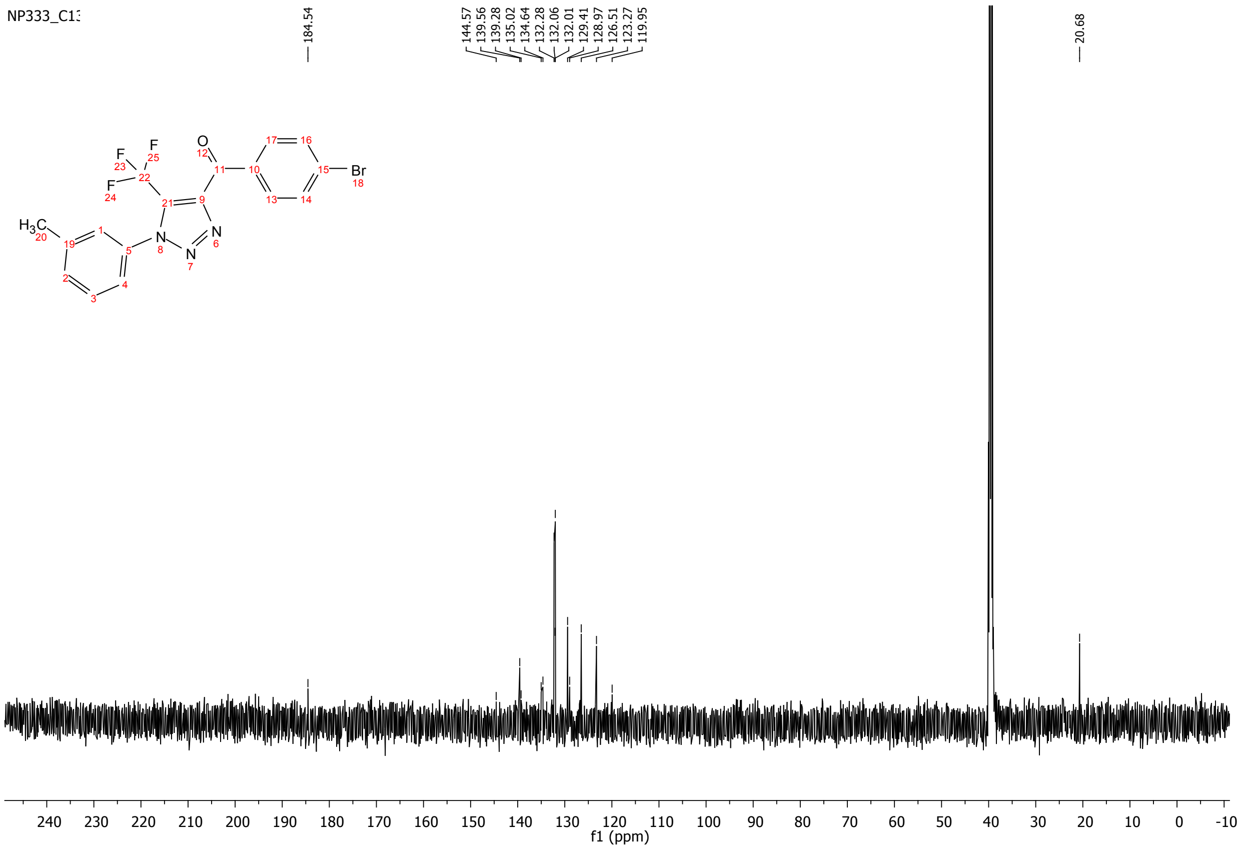

Figure S2. ^{13}C NMR (4-Bromophenyl)(1-(m-tolyl)-5-(trifluoromethyl)-1H-1,2,3-triazol-4-yl)methanone **3a**.

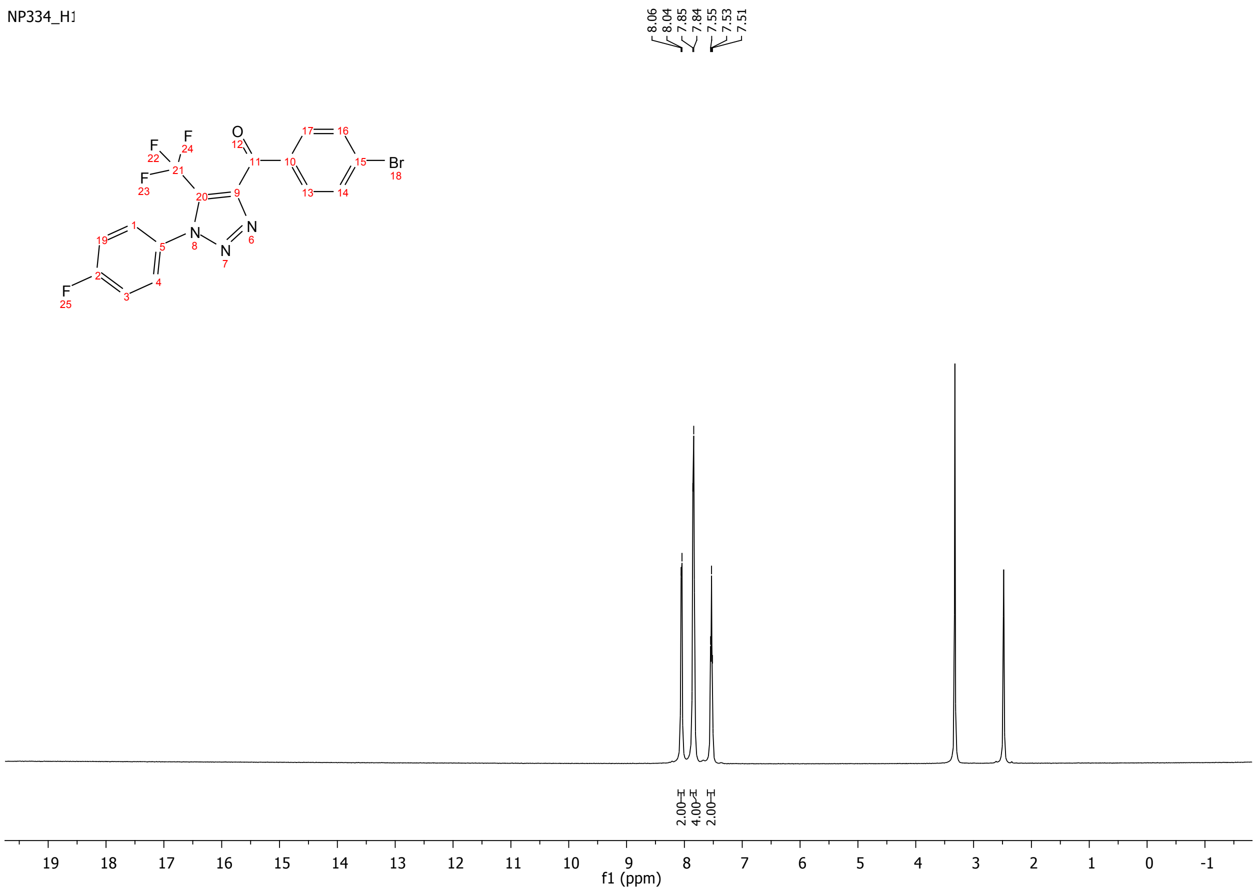

Figure S3. ^{1}H NMR (4-Bromophenyl)(1-(4-fluorophenyl)-5-(trifluoromethyl)-1H-1,2,3-triazol-4-yl)methanone **3b.**

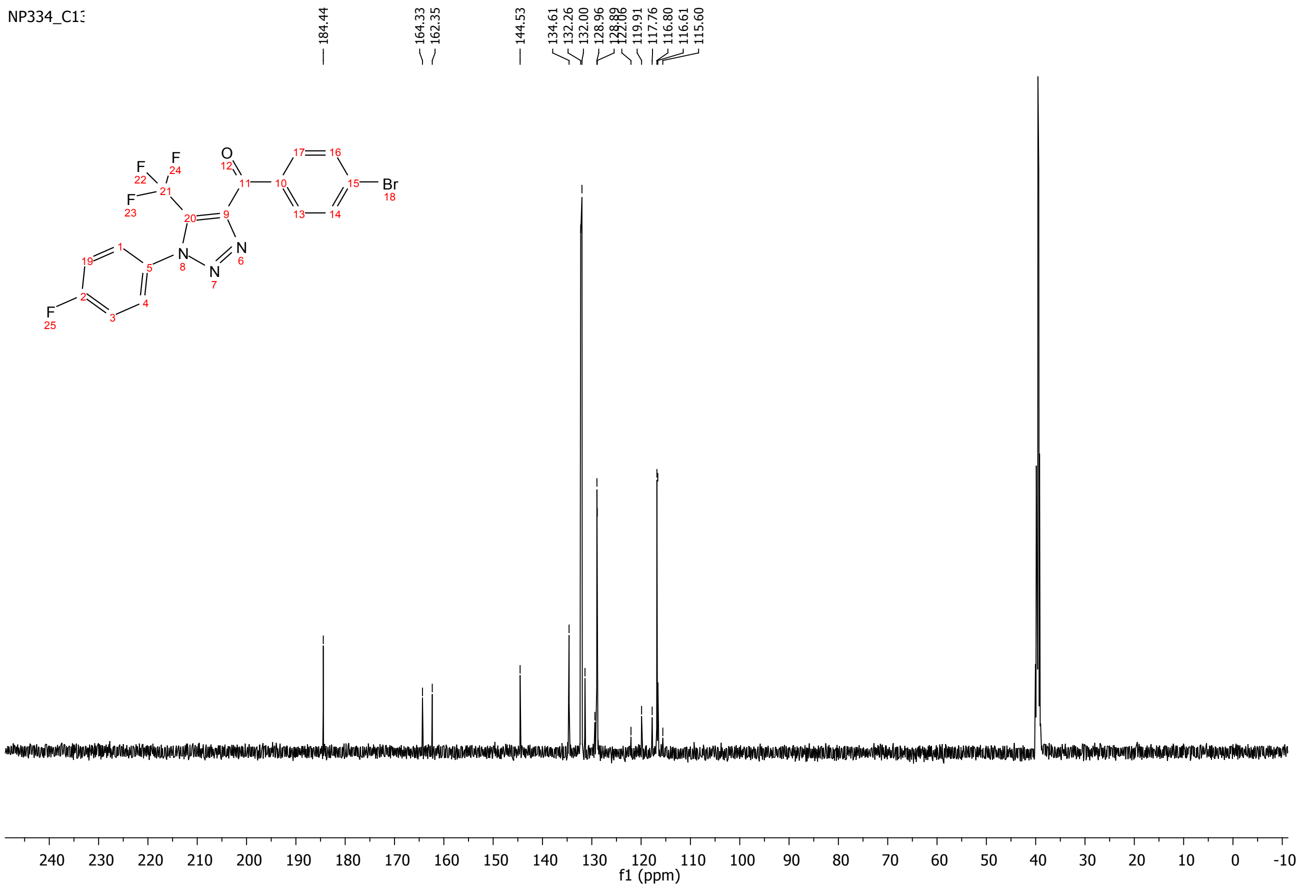

Figure S4. ^{13}C NMR (4-Bromophenyl)(1-(4-fluorophenyl)-5-(trifluoromethyl)-1H-1,2,3-triazol-4-yl)methanone **3b.**

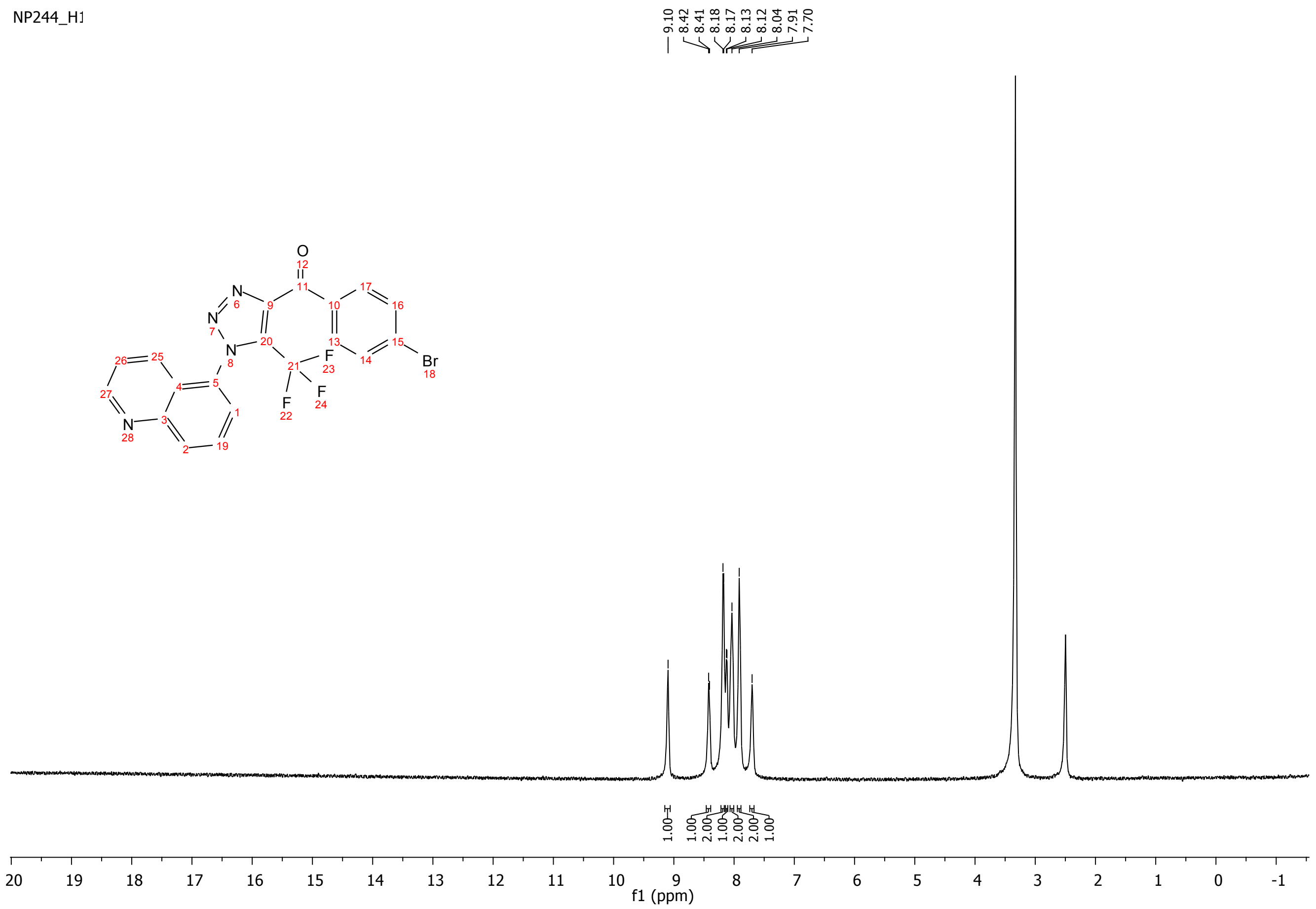

Figure S5. ^{1}H NMR (4-bromophenyl)(1-(quinolin-5-yl)-5-(trifluoromethyl)-1H-1,2,3-triazol-4-yl)methanone **3c.**

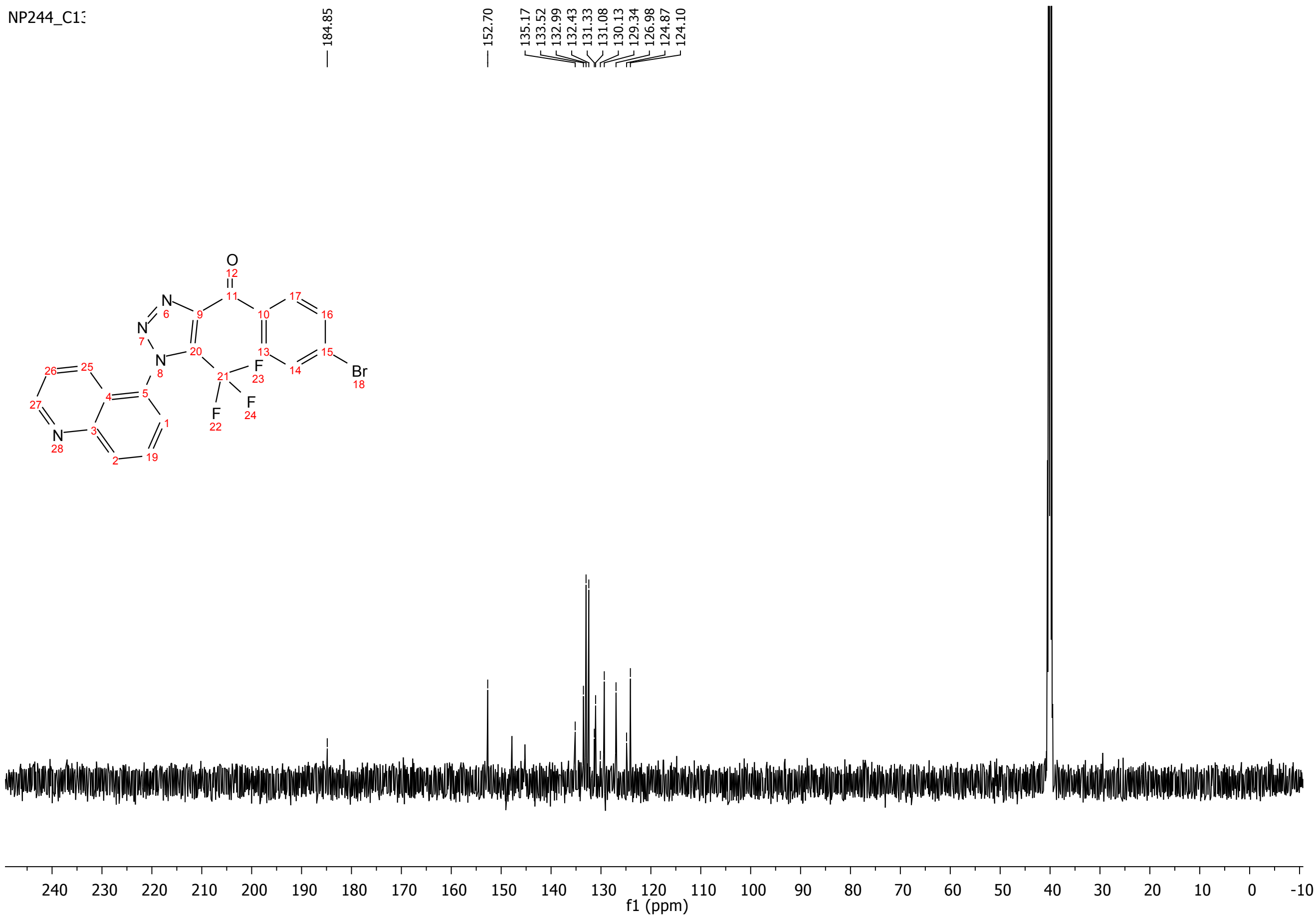

Figure S6. ^{13}C NMR (4-bromophenyl)(1-(quinolin-5-yl)-5-(trifluoromethyl)-1H-1,2,3-triazol-4-yl)methanone **3c.**

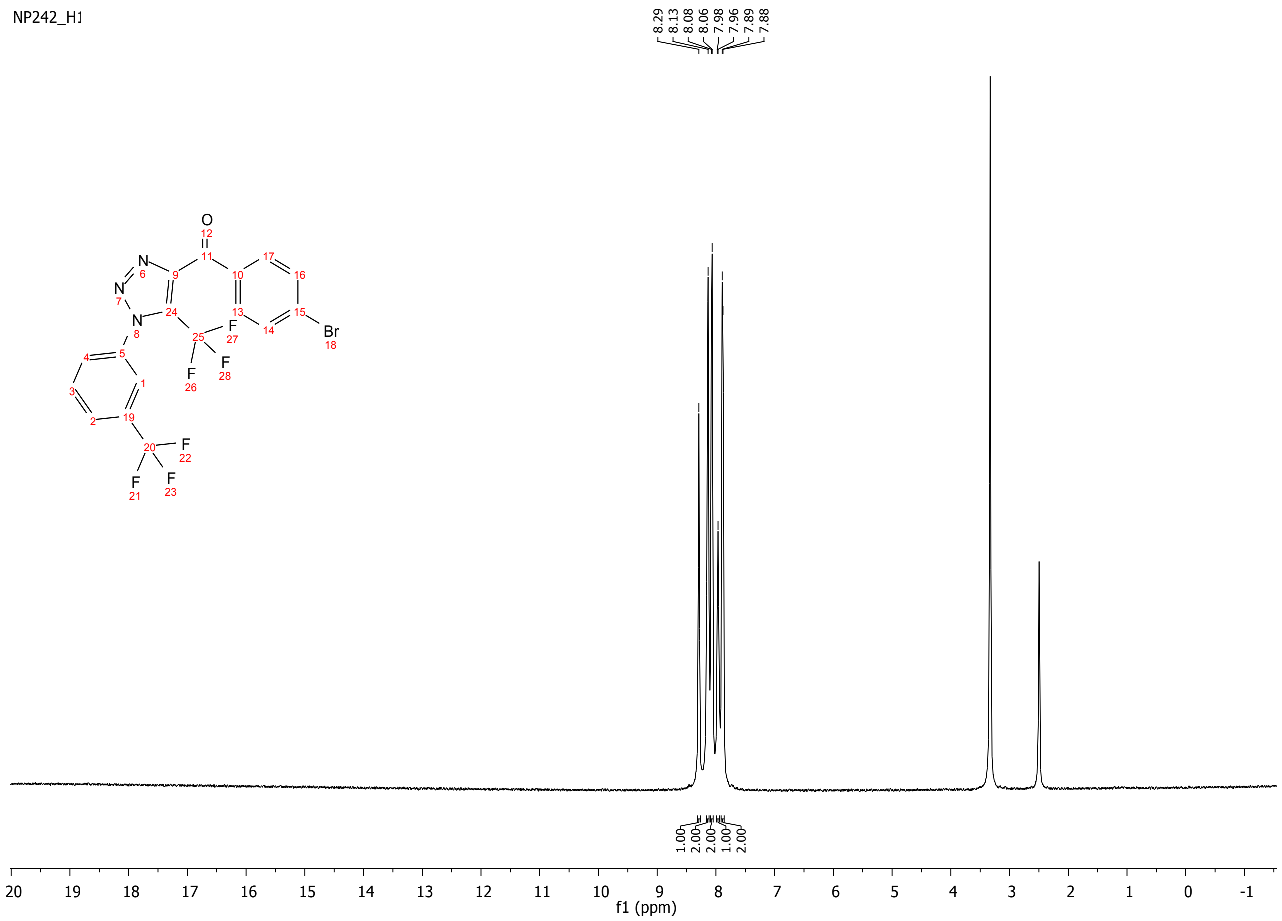

Figure S7. ^{1}H NMR (4-Bromophenyl)(5-(trifluoromethyl)-1-(3-(trifluoromethyl)phenyl)-1H-1,2,3-triazol-4-yl)methanone **3d.**

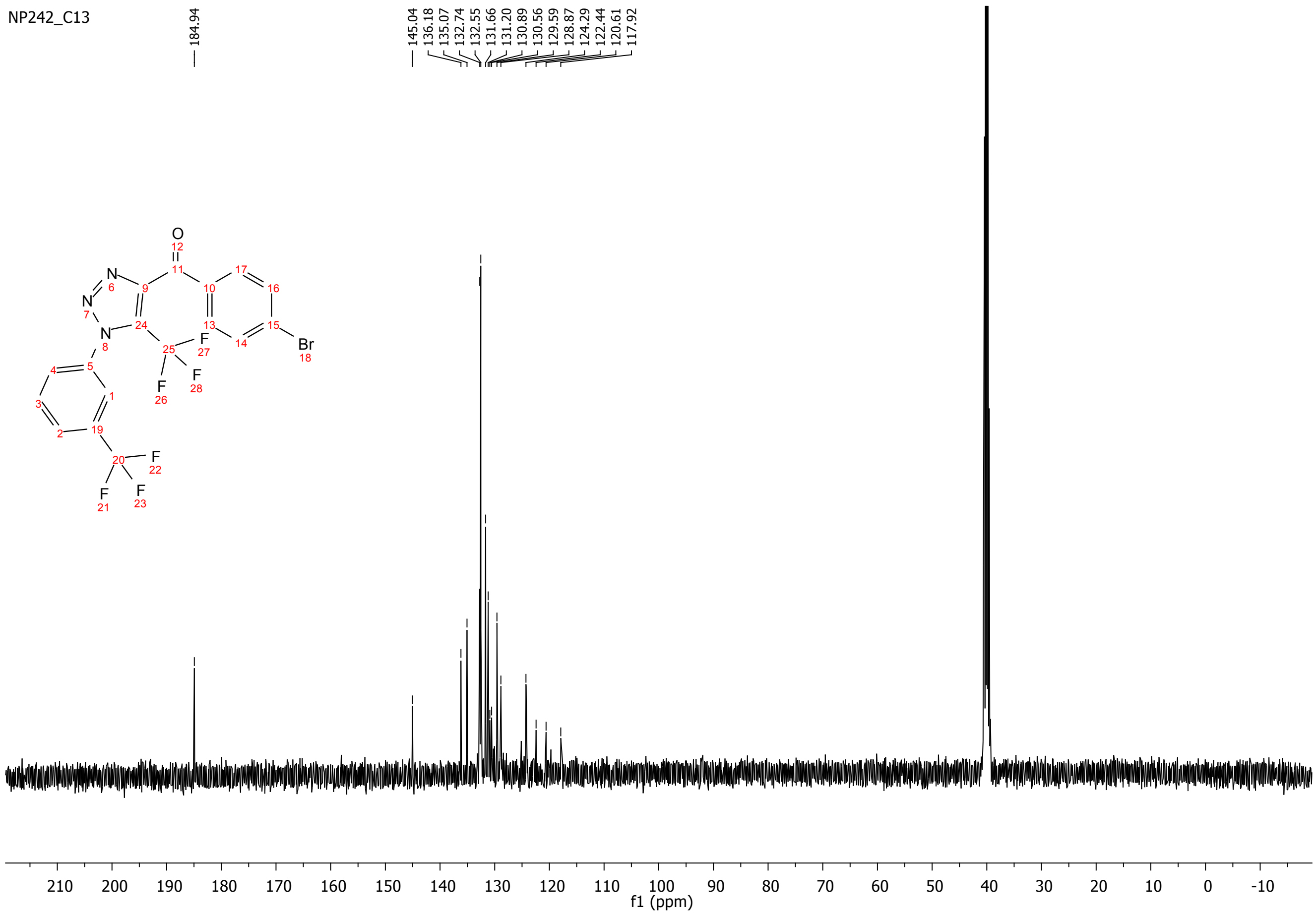

Figure S8. ^{13}C NMR (4-Bromophenyl)(5-(trifluoromethyl)-1-(3-(trifluoromethyl)phenyl)-1H-1,2,3-triazol-4-yl)methanone **3d.**

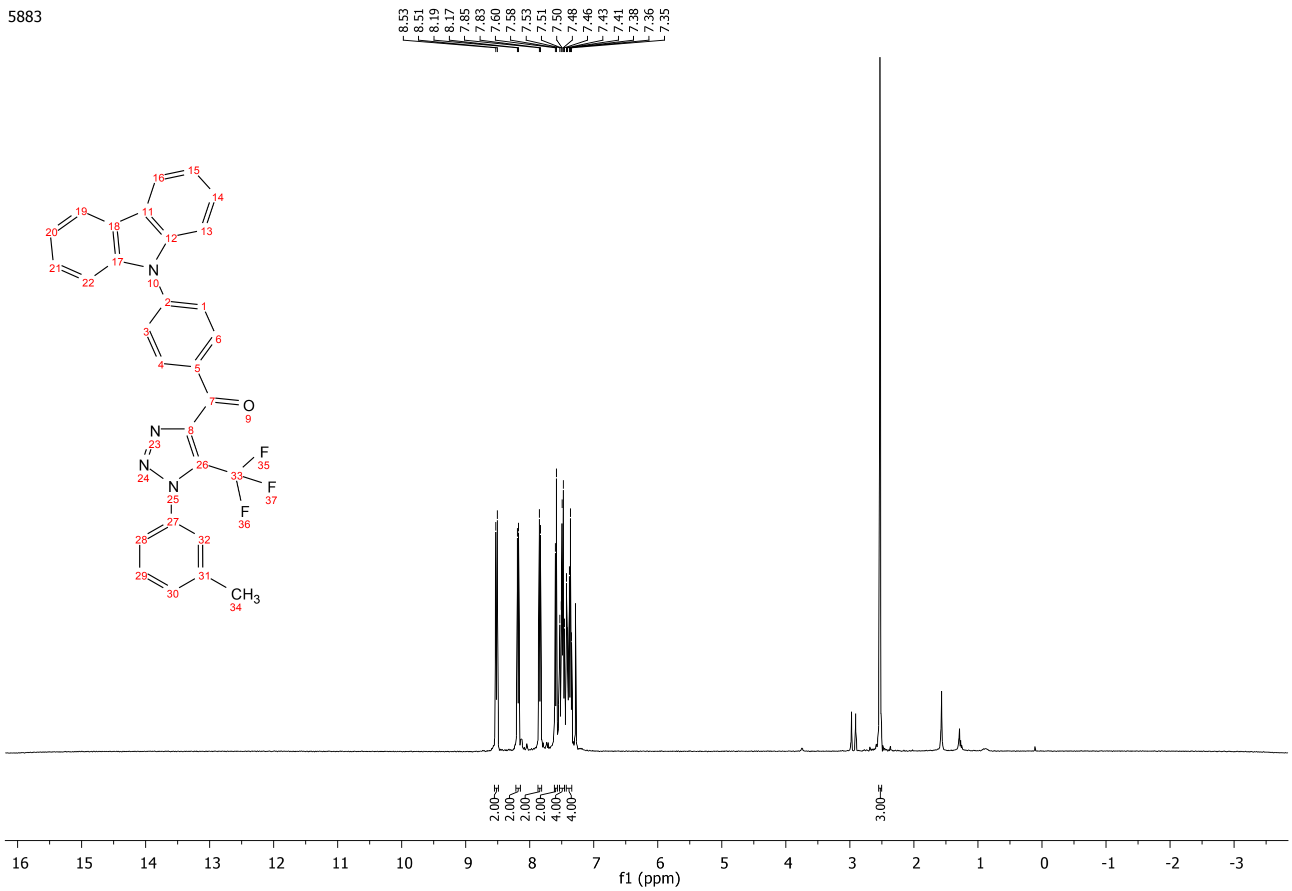

Figure S9. ^{1}H NMR (4-(9H-Carbazol-9-yl)phenyl)(1-(m-tolyl)-5-(trifluoromethyl)-1H-1,2,3-triazol-4-yl)methanone **5a**.

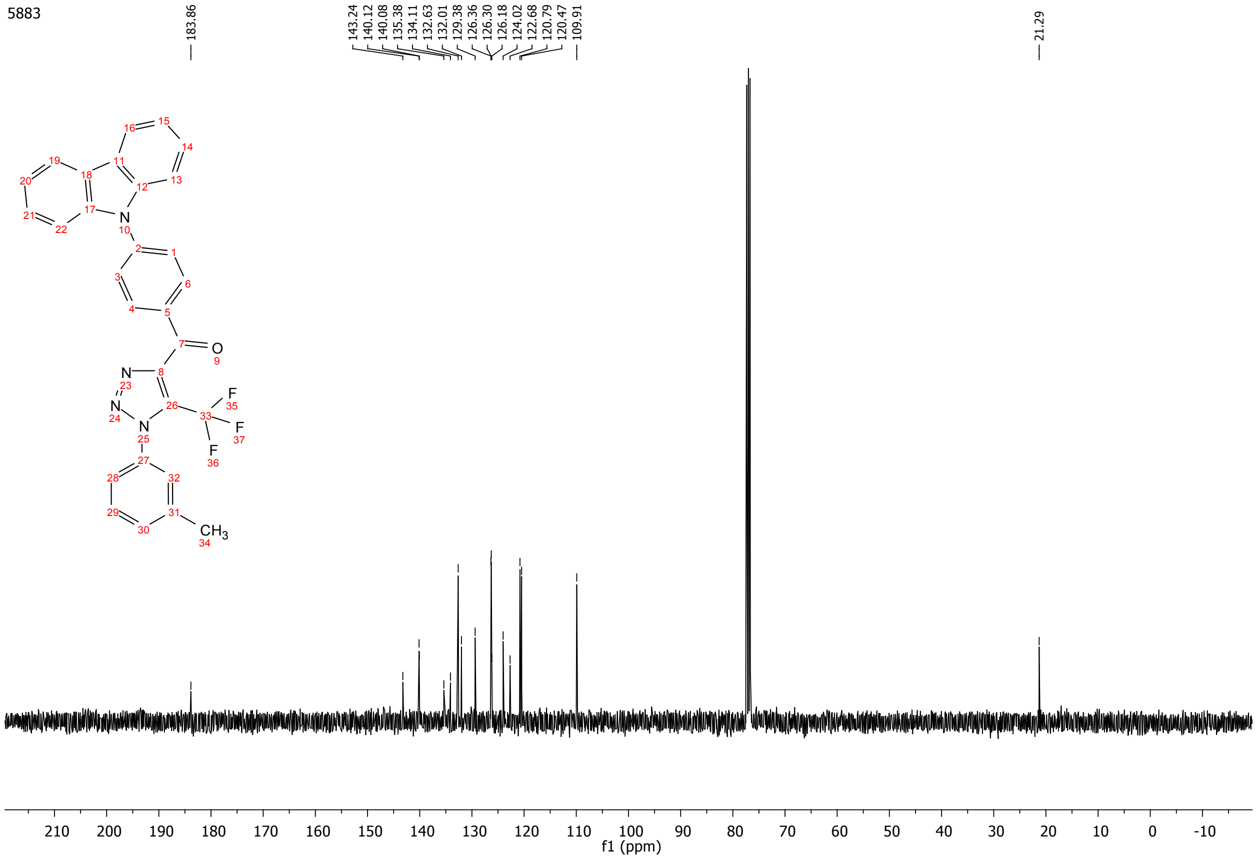

Figure S10. ^{13}C NMR (4-(9H-Carbazol-9-yl)phenyl)(1-(m-tolyl)-5-(trifluoromethyl)-1H-1,2,3-triazol-4-yl)methanone **5a**.

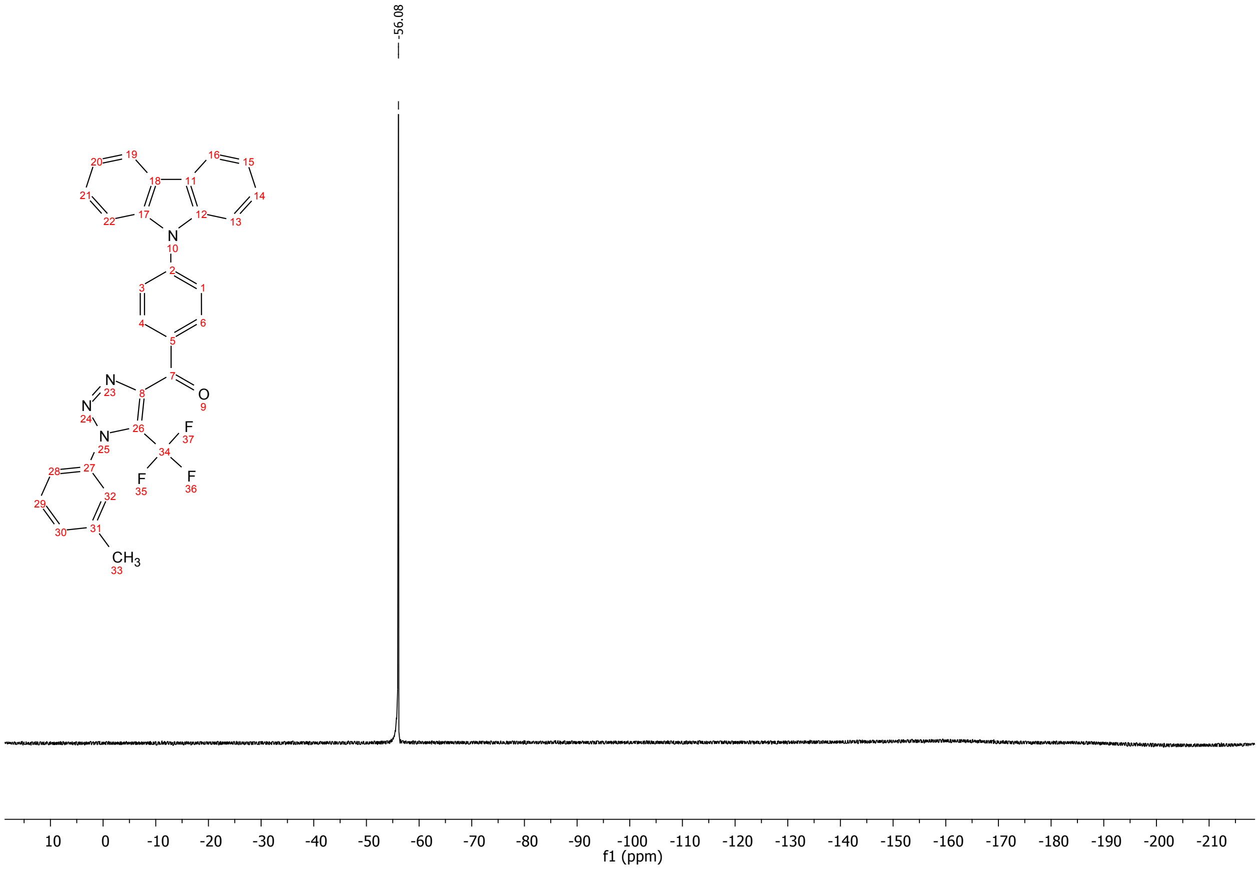

Figure S11. ^{19}F NMR (4-(9H-Carbazol-9-yl)phenyl)(1-(m-tolyl)-5-(trifluoromethyl)-1H-1,2,3-triazol-4-yl)methanone **5a**.

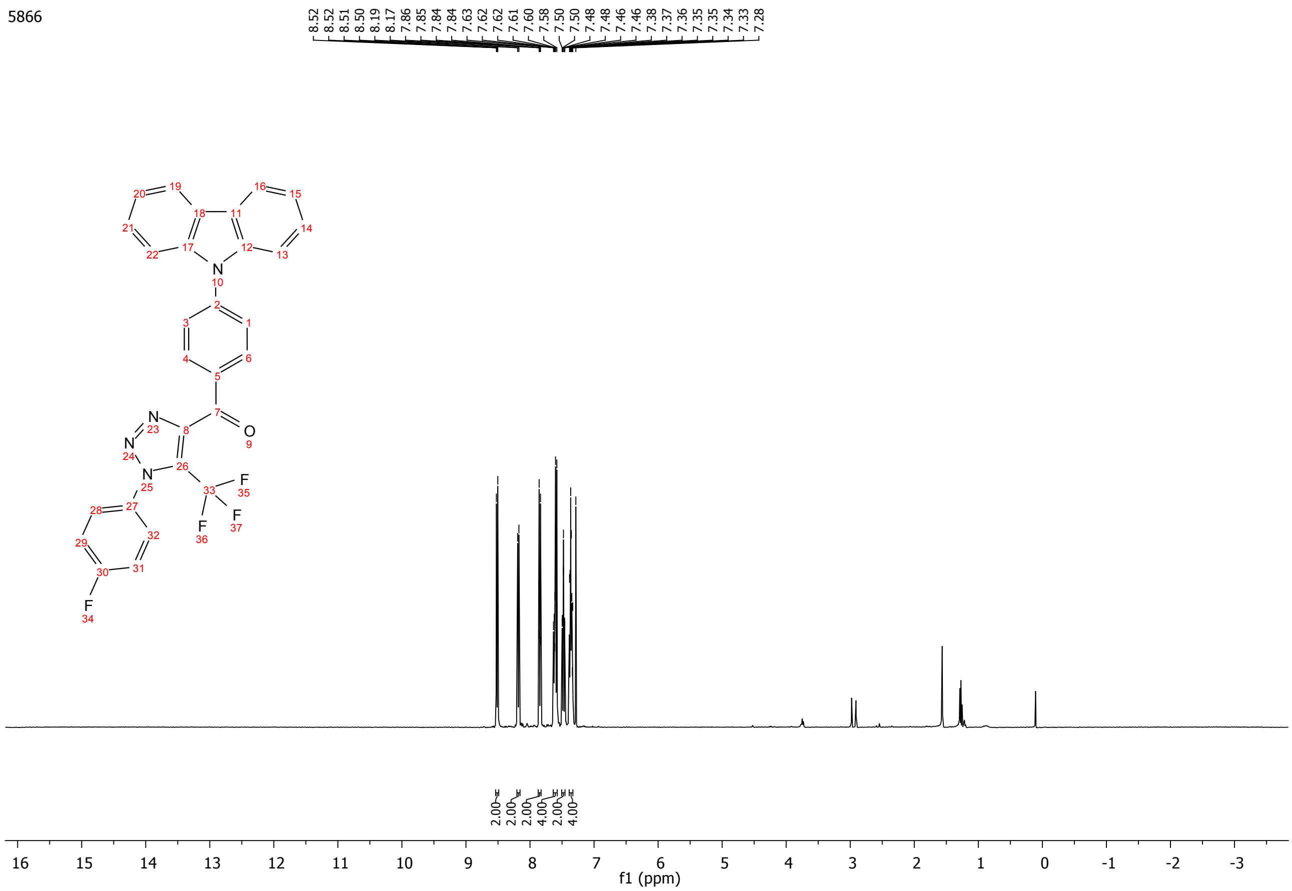

Figure S12. ^{1}H NMR (4-(9H-carbazol-9-yl)phenyl)(1-(4-fluorophenyl)-5-(trifluoromethyl)-1H-1,2,3-triazol-4-yl)methanone **5b**.

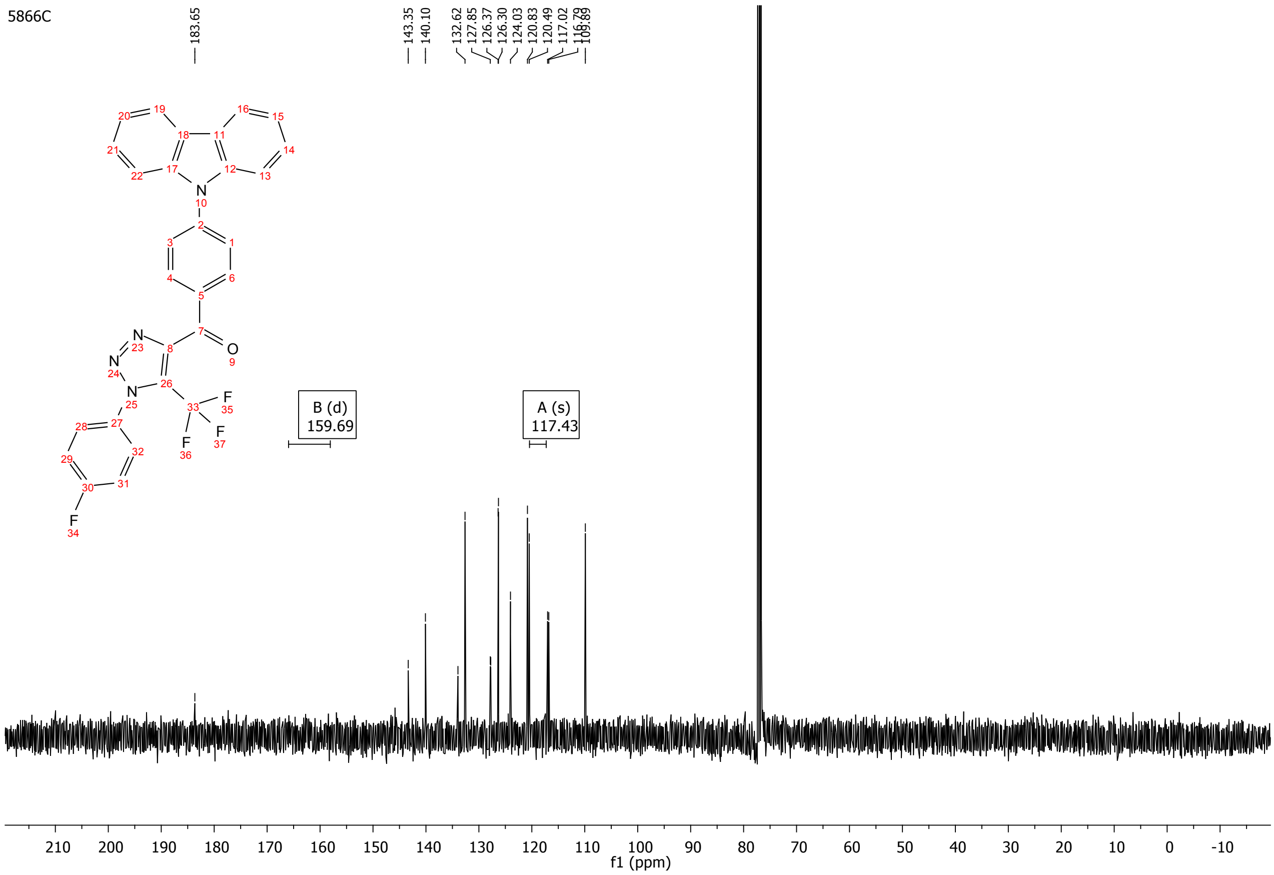

Figure S13. ^{1}H NMR (4-(9H-carbazol-9-yl)phenyl)(1-(4-fluorophenyl)-5-(trifluoromethyl)-1H-1,2,3-triazol-4-yl)methanone **5b**.

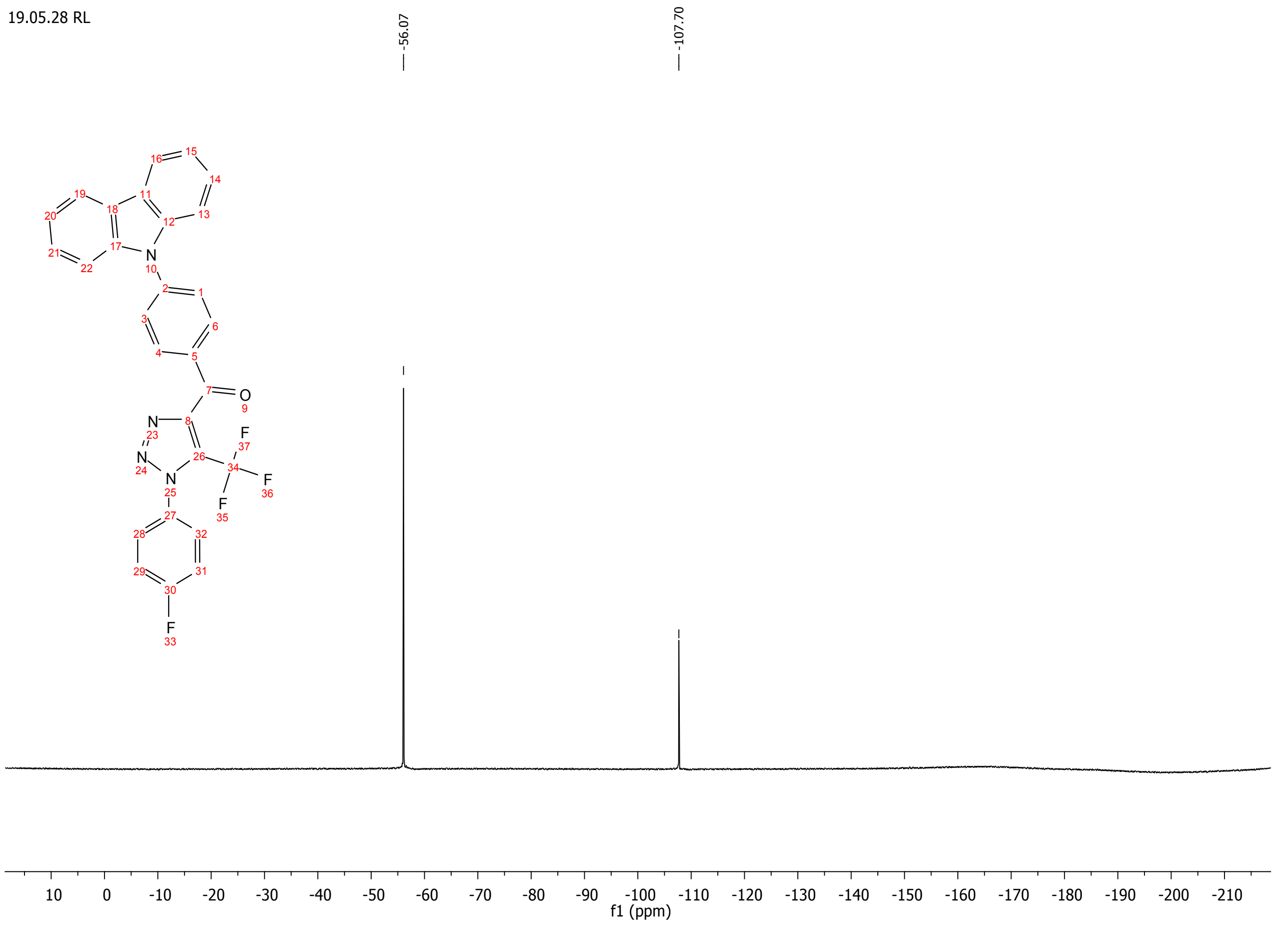

Figure S14. ^{19}F NMR(4-(9H-carbazol-9-yl)phenyl)(1-(4-fluorophenyl)-5-(trifluoromethyl)-1H-1,2,3-triazol-4-yl)methanone **5b**.

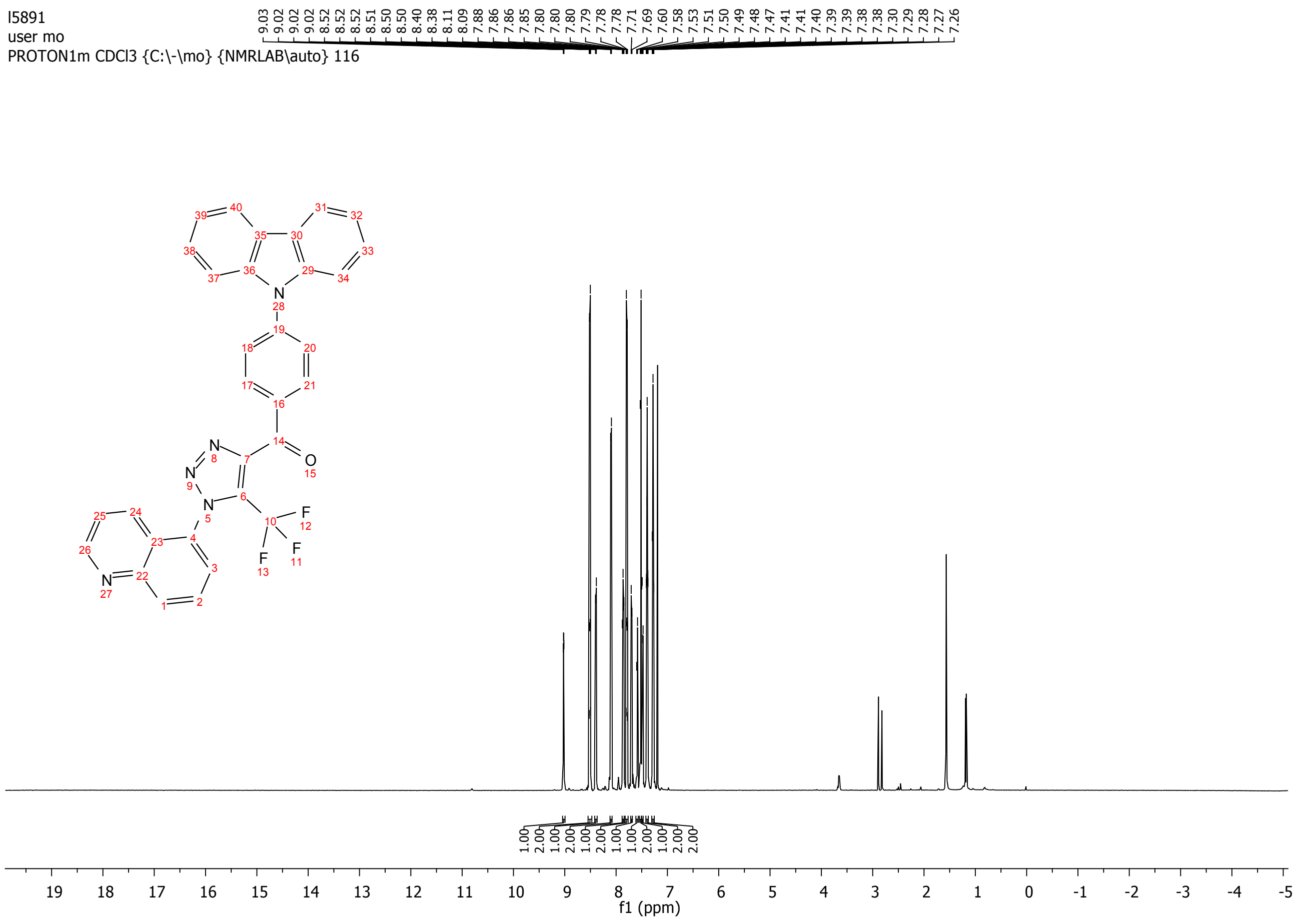

Figure S15. ^{1}H NMR (4-(9H-carbazol-9-yl)phenyl)(1-(quinolin-5-yl)-5-(trifluoromethyl)-1H-1,2,3-triazol-4-yl)methanone **5c**.

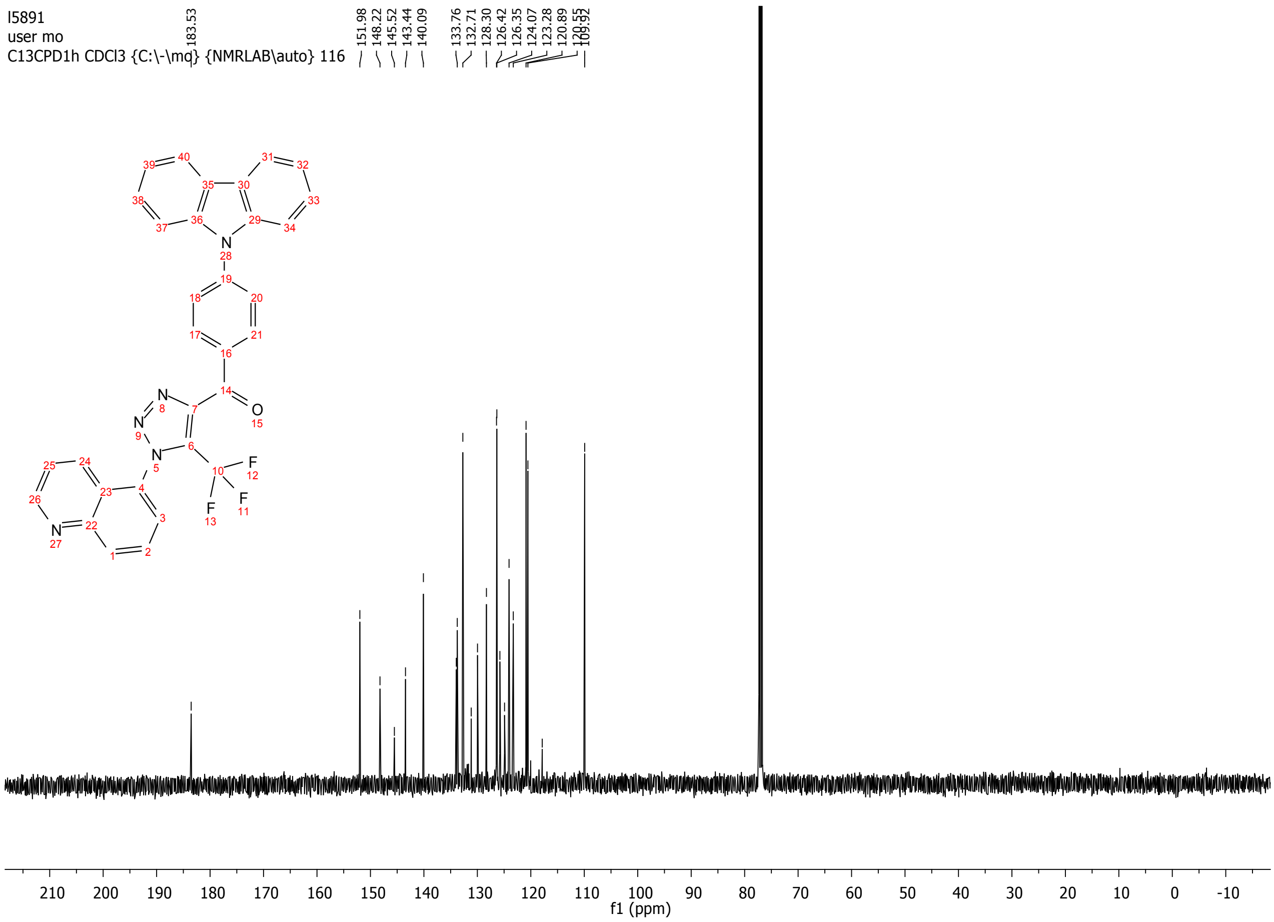

Figure S16. ^{13}C NMR(4-(9H-carbazol-9-yl)phenyl)(1-(quinolin-5-yl)-5-(trifluoromethyl)-1H-1,2,3-triazol-4-yl)methanone **5c**.

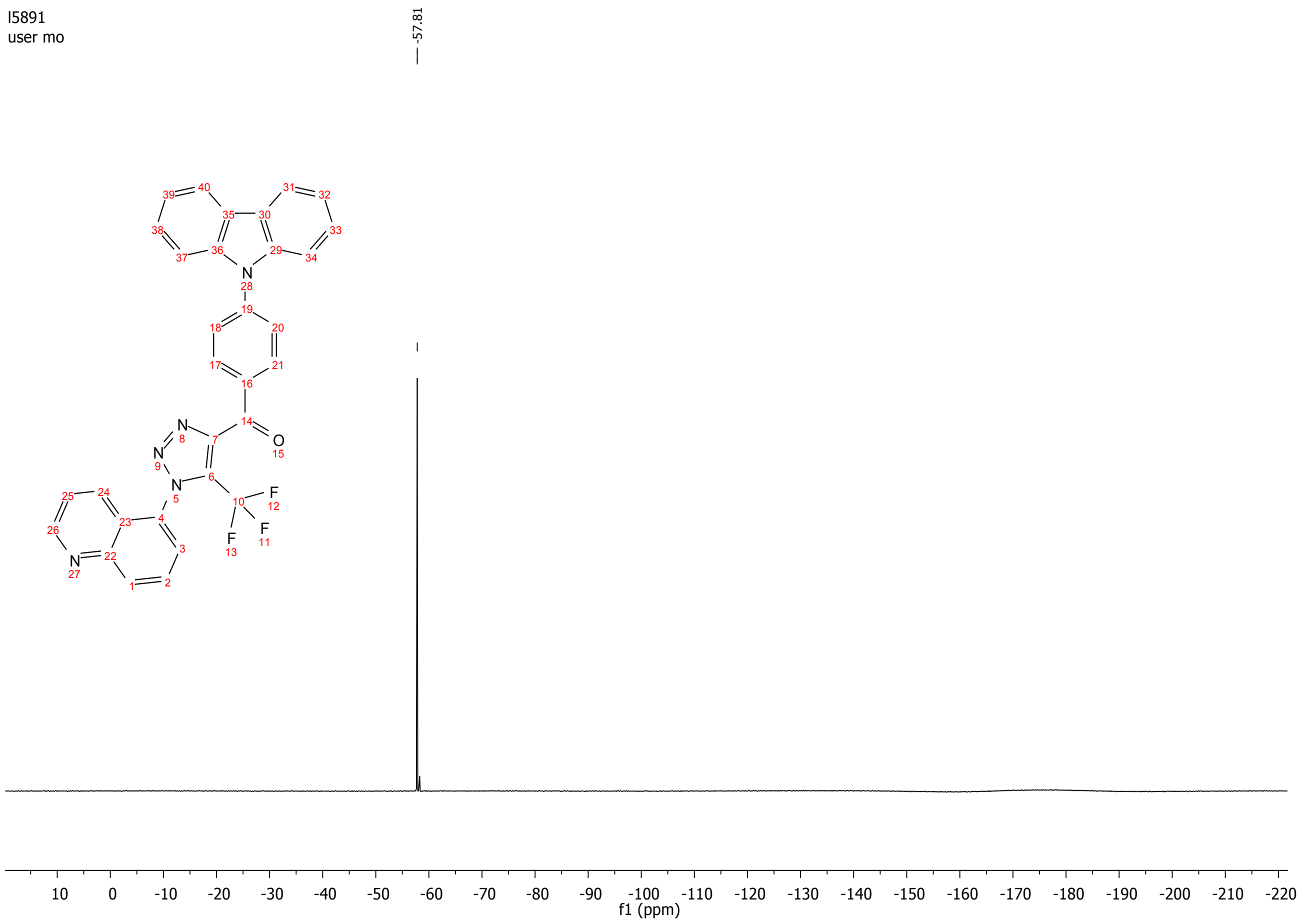

Figure S17. ^{19}F NMR(4-(9H-carbazol-9-yl)phenyl)(1-(quinolin-5-yl)-5-(trifluoromethyl)-1H-1,2,3-triazol-4-yl)methanone **5c**.

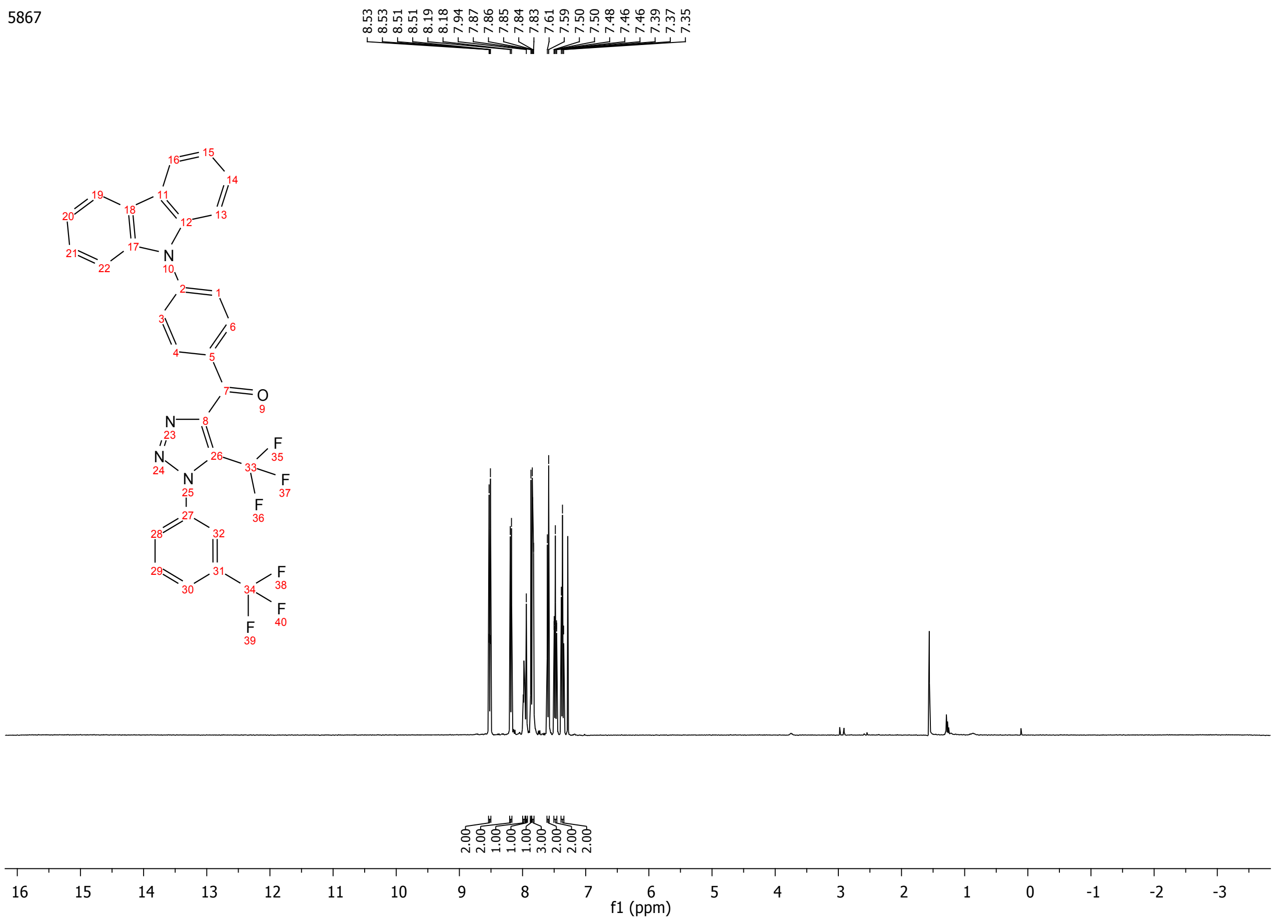

Figure S18. ^{1}H NMR (4-(9H-carbazol-9-yl)phenyl)(5-(trifluoromethyl)-1-(3-(trifluoromethyl)phenyl)-1H-1,2,3-triazol-4-yl)methanone **5d.**

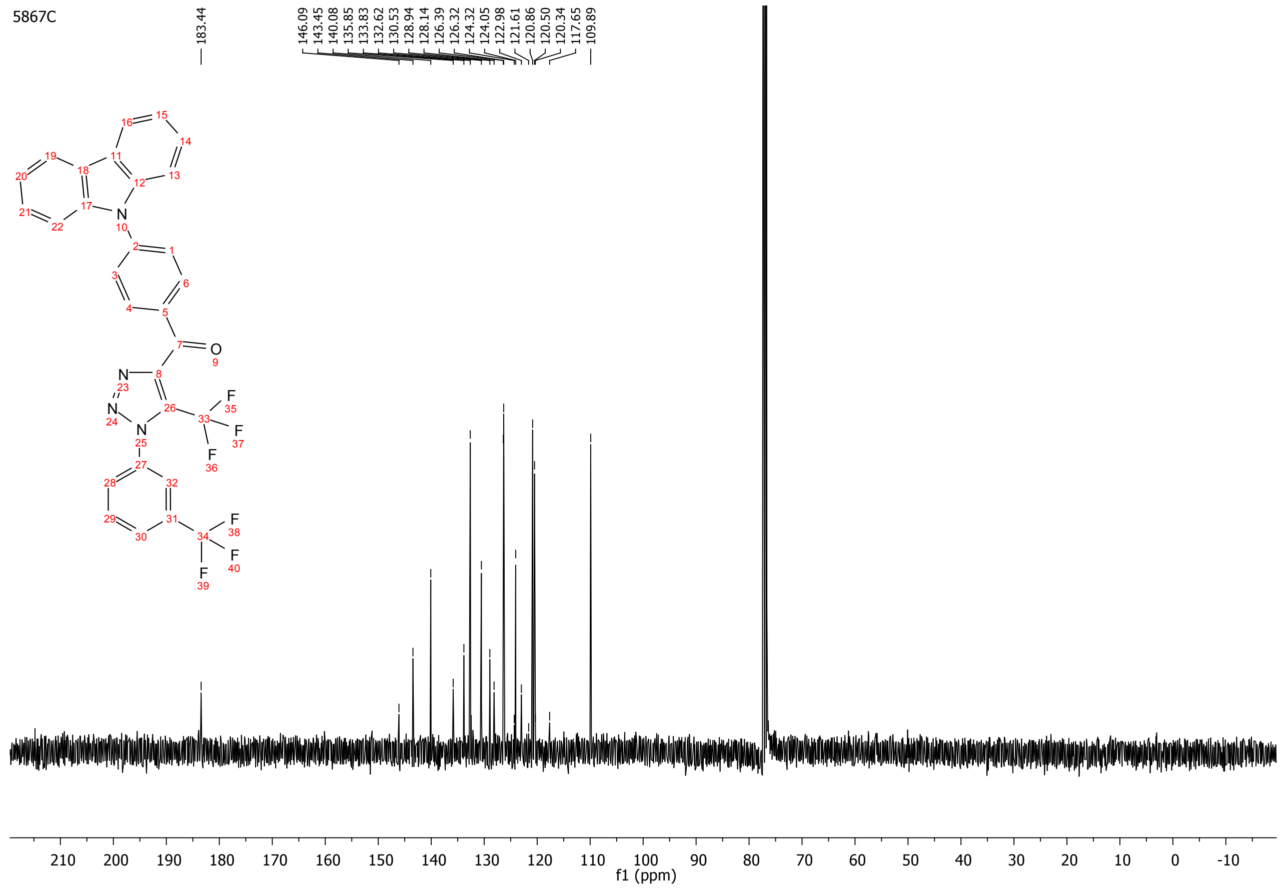

Figure S19. ^{13}C NMR (4-(9H-carbazol-9-yl)phenyl)(5-(trifluoromethyl)-1-(3-(trifluoromethyl)phenyl)-1H-1,2,3-triazol-4-yl)methanone **5d.**

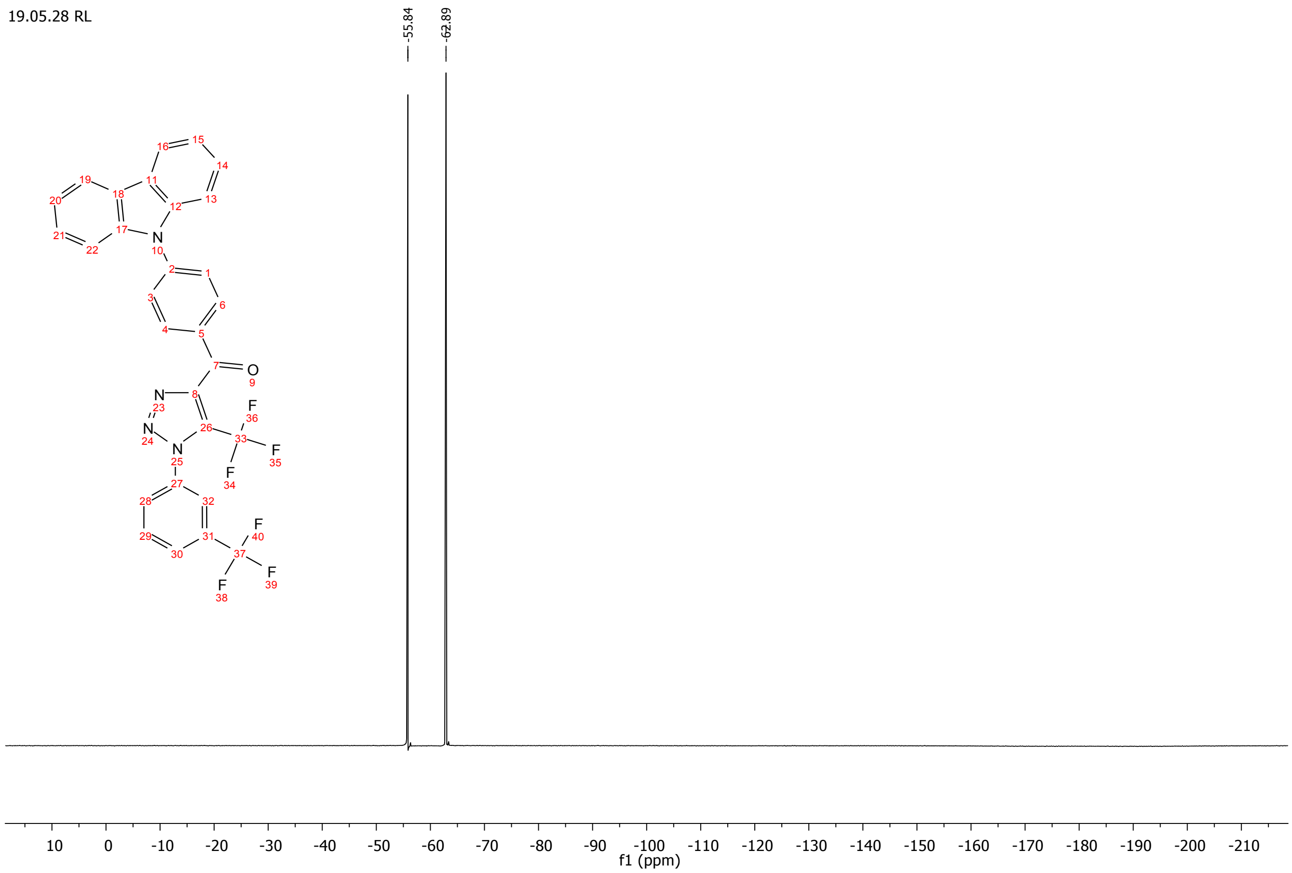

Figure S20. ^{19}F NMR (4-(9H-carbazol-9-yl)phenyl)(5-(trifluoromethyl)-1-(3-(trifluoromethyl)phenyl)-1H-1,2,3-triazol-4-yl)methanone **5d.**

Table S1. Crystal data and structure refinements of compound **5b**.

**Formula**	C_{28} H_{16} F_4 N_4 O
**Molecular weight**	500.45
**Crystal system**	triclinic
**Space group**	P -1
**a (Å)**	7.9155 (13)
**b (Å)**	9.6147 (14)
**c (Å)**	14.393 (2)
***α* (deg)**	99.94 (3)
***β* (deg)**	93.37 (3)
***γ* (deg)**	92.25 (3)
**Cell volume, (Å^3)**	1075.71
**Z, Z'**	2; 0
**μ (mm^{-1})**	1.026
**F (000)**	512.0

Table S2. Characterization of electronic transitions for compound **5a.**

Geometry	HOMO	LUMO
**5a**	**5.45** eV	**2.38** eV

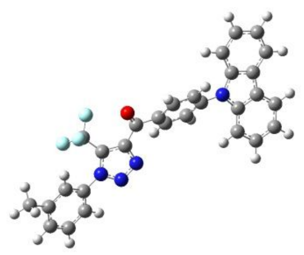 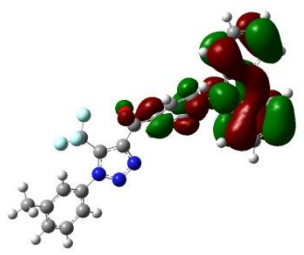 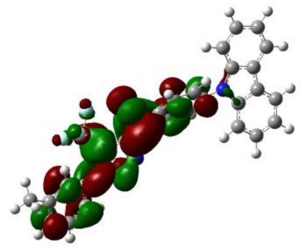

Electronic transition	Transition configuration	Oscillator strength, f	Energy, eV
$S_0 \rightarrow S_1$	HOMO→LUMO (99%)	0.1655	2.7153
$S_0 \rightarrow S_4$	H → L+1 (98%)	0.1219	3.4439
$S_0 \rightarrow S_6$	H-1→ L+7 (5%)<br>H→ L+2 (92%)	0.05823	3.9945
$S_0 \rightarrow S_7$	H-5→ L (6%)<br>H-4→ L (17%)<br>H-3→ L (49%)<br>H-2→ L (22%)	0.2227	4.0609
$S_0 \rightarrow S_{10}$	H-7→ L (5%)<br>H-6→ L (82%)<br>H-4→ L (6%)	0.2132	4.2682

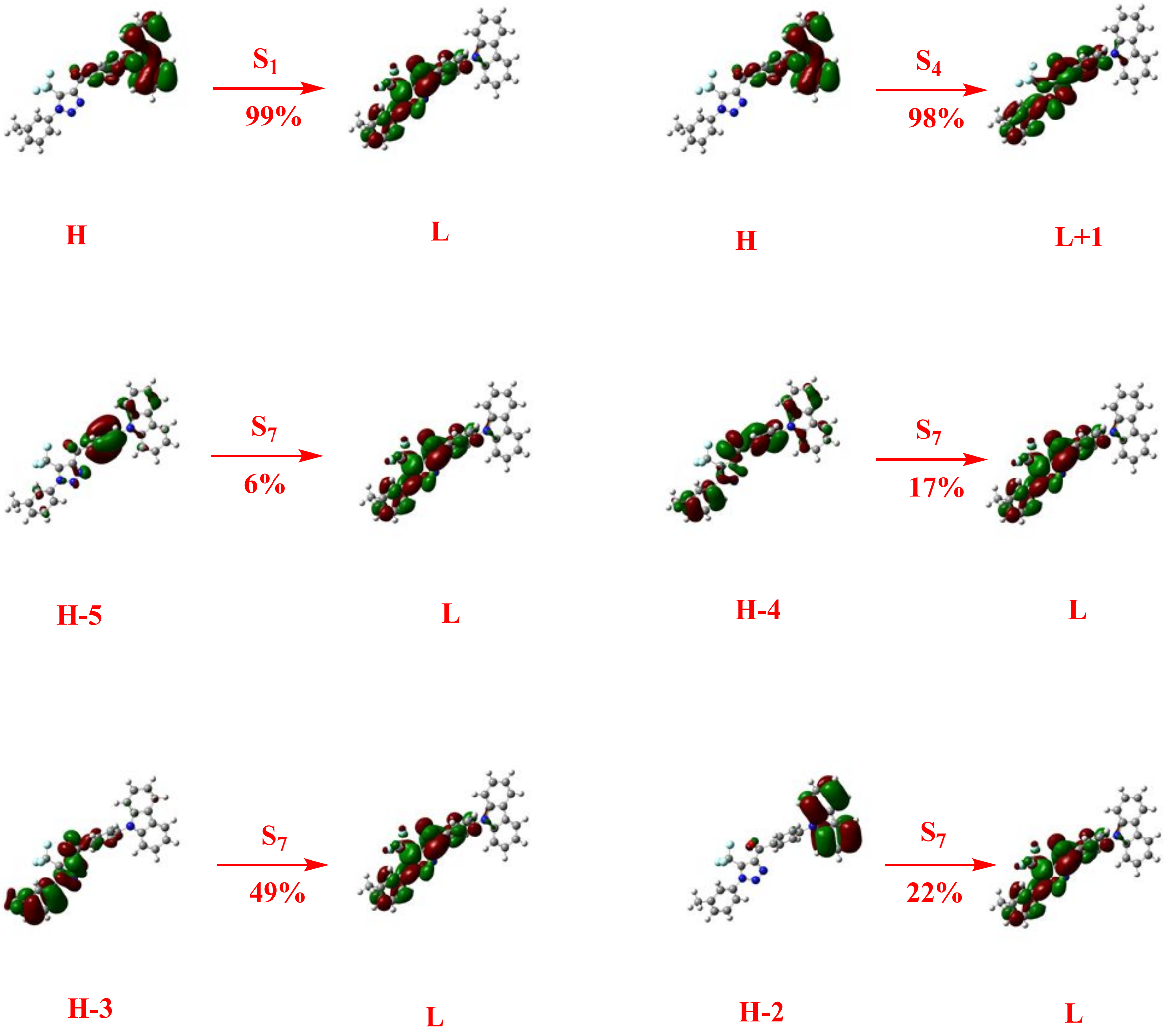

Figure S21. Visualization of electronic transitions for compound **5a**, simulated with Gaussian'16 software.

Table S3. Characterization of electronic transitions for compound **5b.**

**Geometry**	**HOMO**	**LUMO**
**5b** 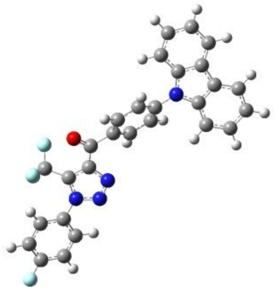	**5.49** eV 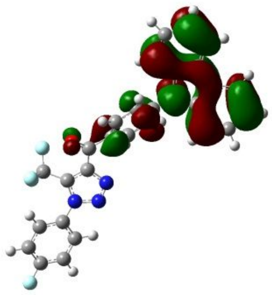	**2.47** eV 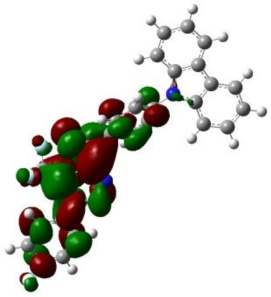

**Electronic transition**	**Transition configuration**	**Oscillator strength, f**	**Energy, eV**
$S_0 \rightarrow S_1$	HOMO→LUMO (99%)	0.1576	2.6668
$S_0 \rightarrow S_4$	H-3 → L (4%)<br>H→ L+1 (93%)	0.1151	3.3861
$S_0 \rightarrow S_8$	H-6→ L (5%)<br>H-5→ L (7%)<br>H-4→ L(61%)<br>H-3→ L (15%)<br>H→ L+4 (5%)	0.3736	4.0990

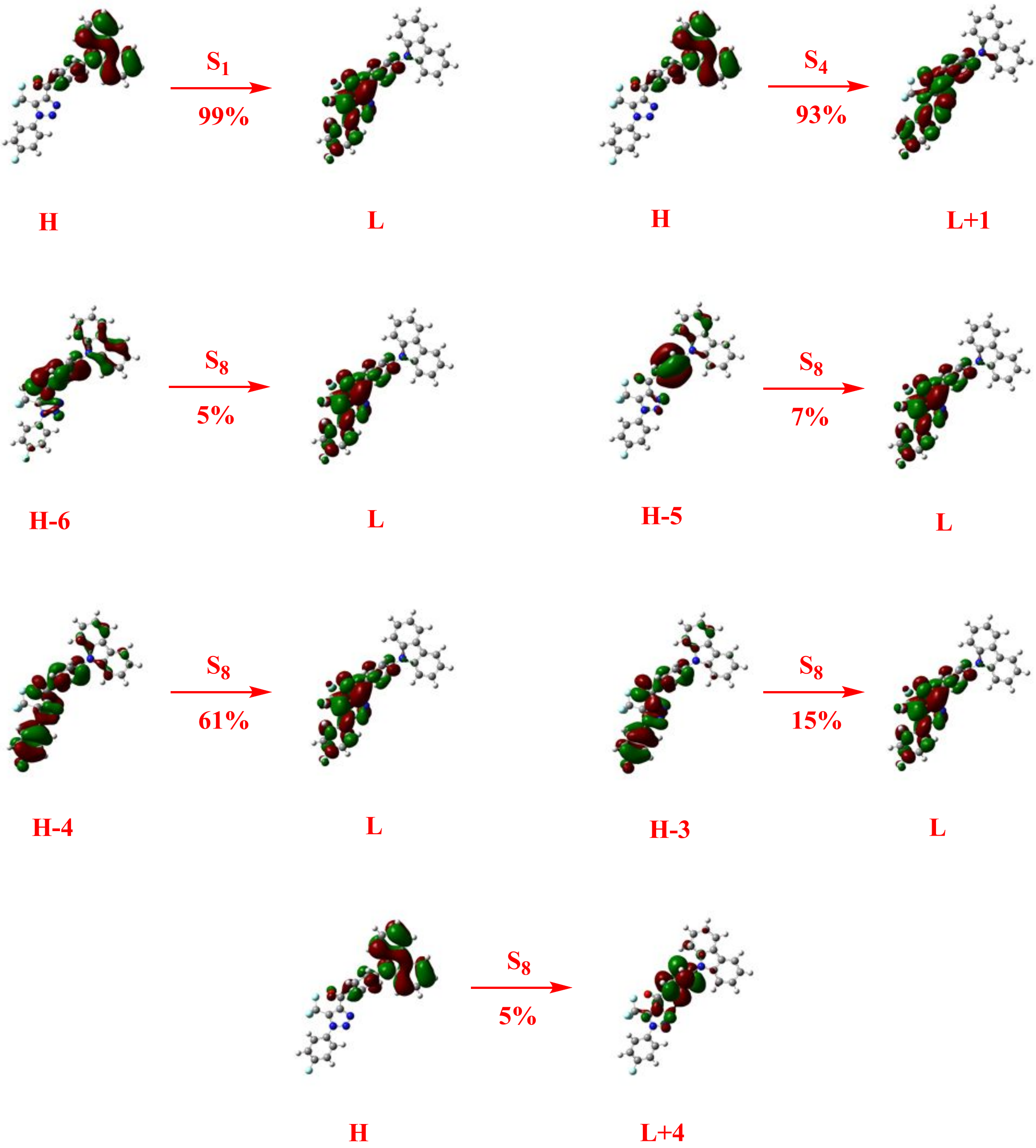

Figure S22. Visualization of electronic transitions for compound **5b**, simulated with Gaussian'16 software.

Table S4. Characterization of electronic transitions for compound **5c.**

Geometry	HOMO	LUMO
**5c**	**5,46** eV	**2,61** eV
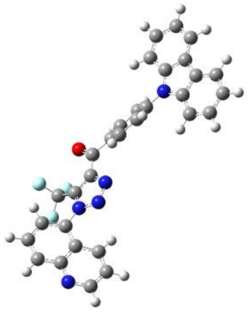	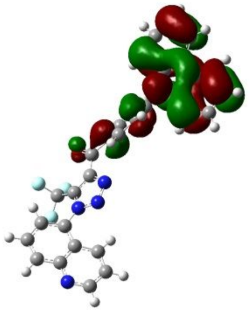	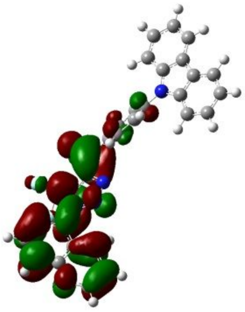

Electronic transition	Transition configuration	Oscillator strength, f	Energy, eV
$S_0 \rightarrow S_1$	H→L (98%)	0,1021	2.5317
$S_0 \rightarrow S_3$	H→L+1(98%)	0.1300	3.0815
$S_0 \rightarrow S_6$	H-2→L (93%)<br>H-2→L+1(2%)	0.3429	3.6351

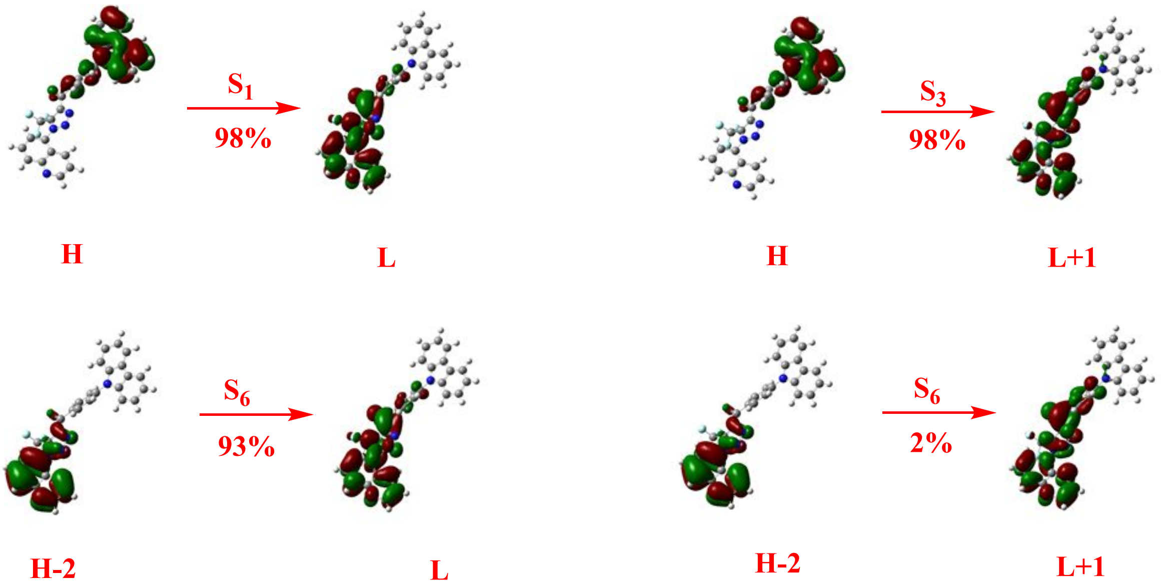

Figure S23. Visualization of electronic transitions for compound **5c**, simulated with Gaussian'16 software.

Table S5. Characterization of electronic transitions for compound **5d.**

Geometry	HOMO	LUMO
**5d** 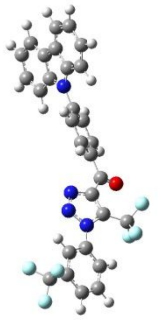	**5,53** eV 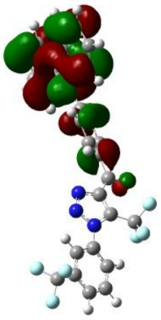	**2,65** eV 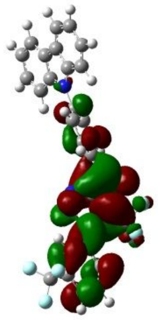

Electronic transition	Transition configuration	Oscillator strength, f	Energy, eV
$S_0 \rightarrow S_1$	H→L (98%)	0,1367	2.5433
$S_0 \rightarrow S_3$	H→L+1(98%)	0.1043	3.2465
$S_0 \rightarrow S_{10}$	H-5→L (34%)<br>H-4→L (27%)<br>H-3→L (30%)	0.2100	4.1383

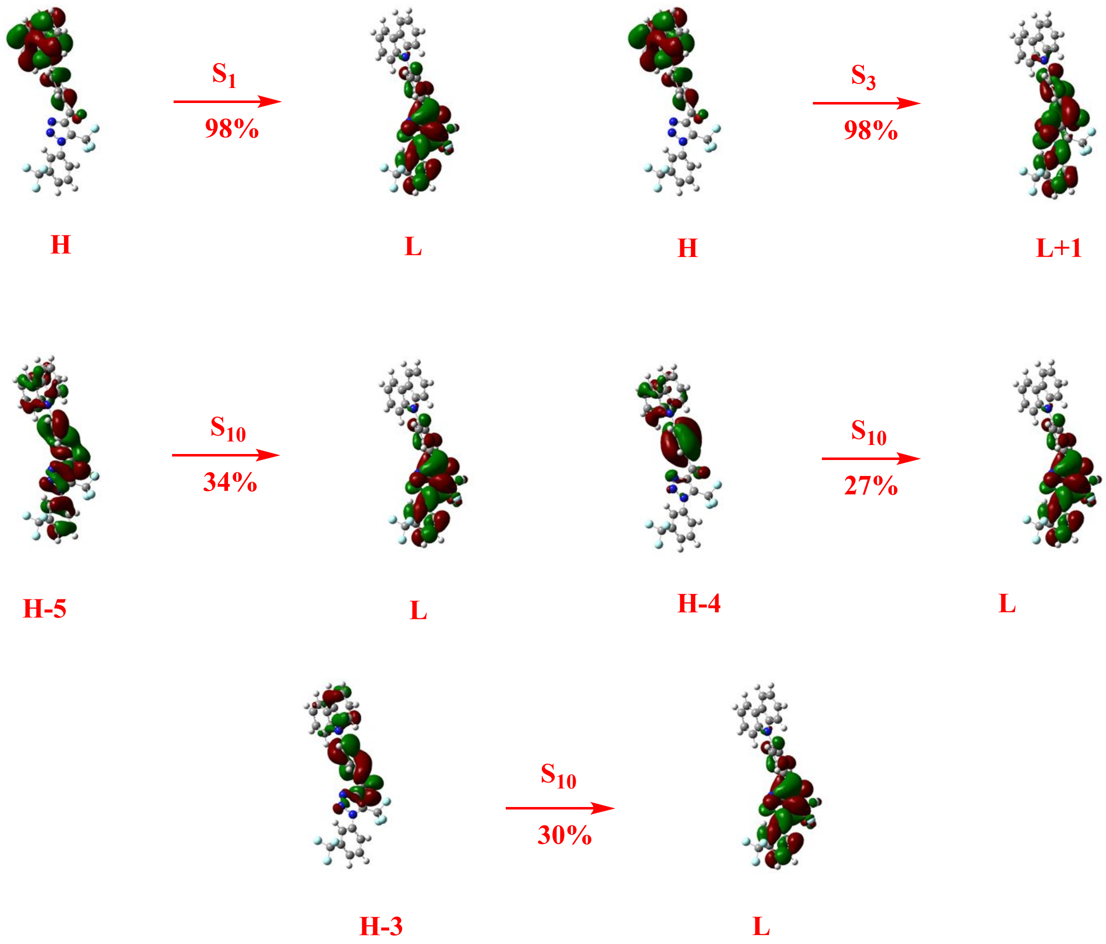

Figure S24. Visualization of electronic transitions for compound **5d**, simulated with Gaussian'16 software.

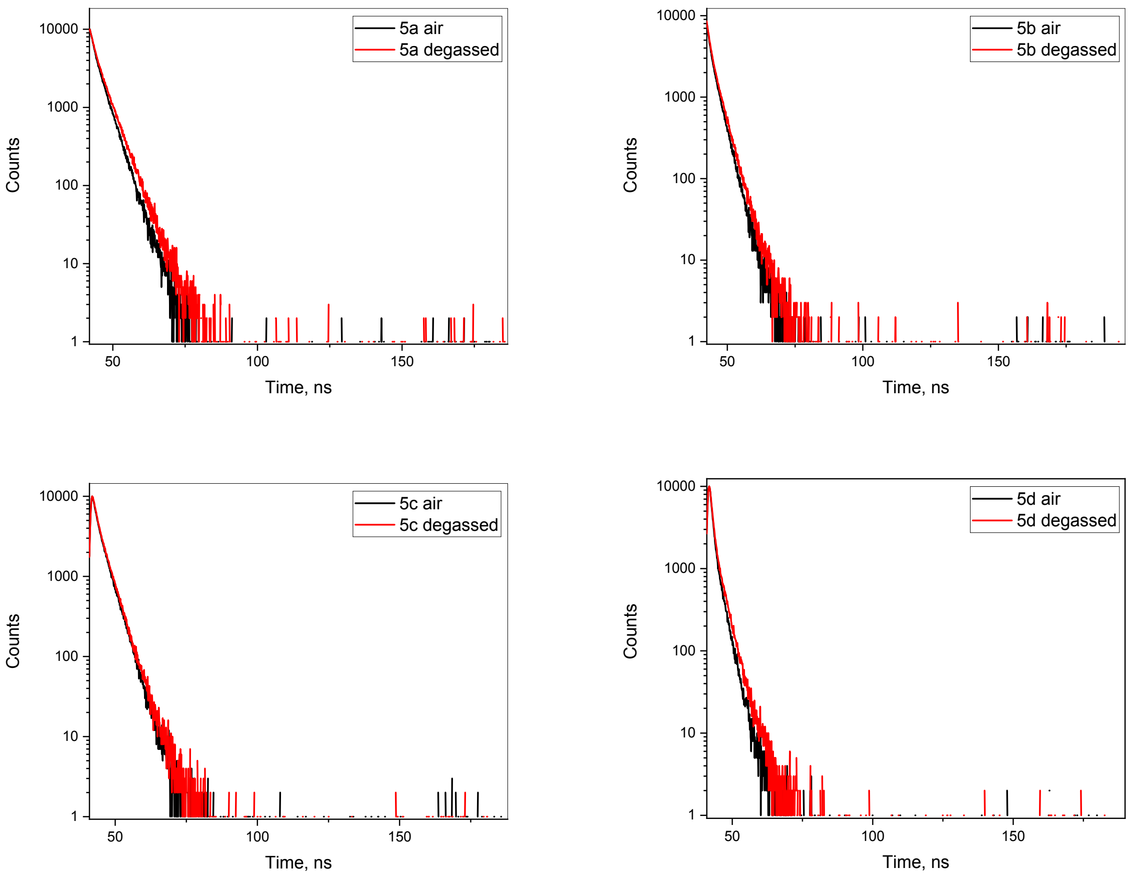

Figure S25. Photoluminescence decay curves of compounds **5a-5d** toluene solutions, degassed and air equilibrated.

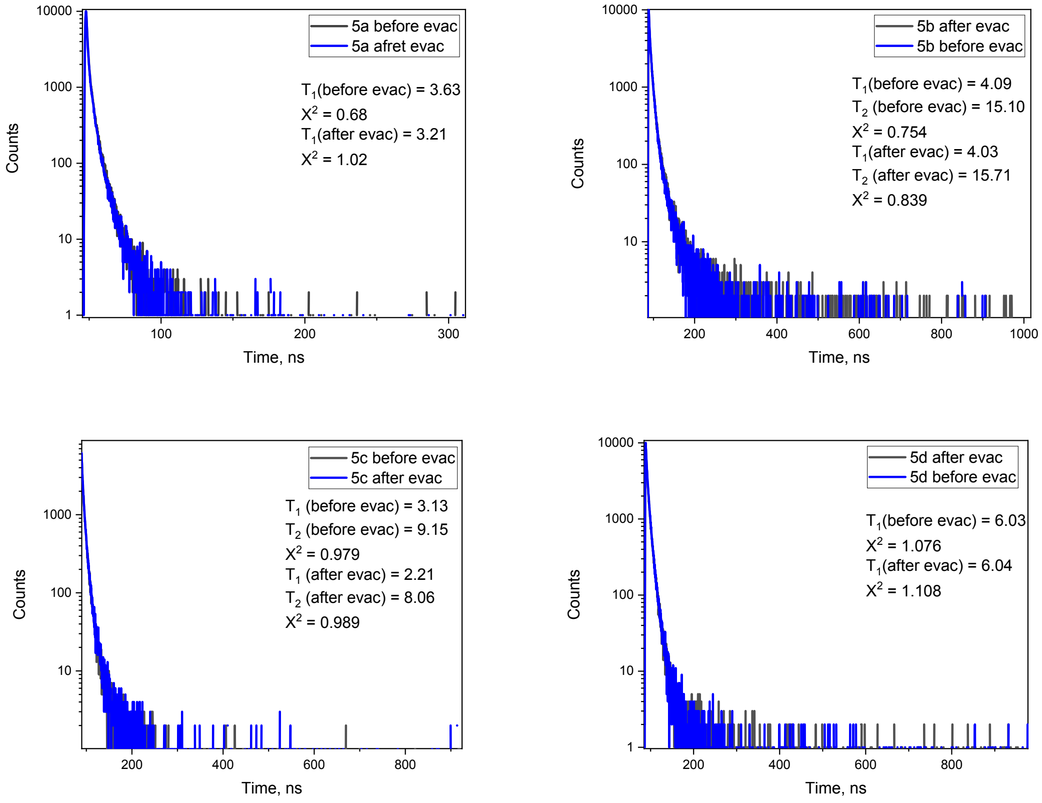

Figure S26. Photoluminescence decay curves of compounds **5a-5d** films before and after evacuation.
